# Green Alternatives as Antimicrobial Agents in Mitigating Periodontal Diseases: A Narrative Review

**DOI:** 10.3390/microorganisms11051269

**Published:** 2023-05-11

**Authors:** Seyed Ali Mosaddad, Ahmed Hussain, Hamid Tebyaniyan

**Affiliations:** 1 Student Research Committee, School of Dentistry, Shiraz University of Medical Sciences, Shiraz 71348-14336, Iran; mosaddad.sa@gmail.com; 2 School of Dentistry, Edmonton Clinic Health Academy, University of Alberta, Edmonton, AB T6G 1C9, Canada; 3 Science and Research Branch, Islimic Azade University, Tehran 14878-92855, Iran

**Keywords:** herbal medicine, plant extracts, periodontal diseases, anti-infective agents, plants

## Abstract

Periodontal diseases and dental caries are the most common infectious oral diseases impacting oral health globally. Oral cavity health is crucial for enhancing life quality since it serves as the entranceway to general health. The oral microbiome and oral infectious diseases are strongly correlated. Gram-negative anaerobic bacteria have been associated with periodontal diseases. Due to the shortcomings of several antimicrobial medications frequently applied in dentistry, the lack of resources in developing countries, the prevalence of oral inflammatory conditions, and the rise in bacterial antibiotic resistance, there is a need for reliable, efficient, and affordable alternative solutions for the prevention and treatment of periodontal diseases. Several accessible chemical agents can alter the oral microbiota, although these substances also have unfavorable symptoms such as vomiting, diarrhea, and tooth discoloration. Natural phytochemicals generated from plants that have historically been used as medicines are categorized as prospective alternatives due to the ongoing quest for substitute products. This review concentrated on phytochemicals or herbal extracts that impact periodontal diseases by decreasing the formation of dental biofilms and plaques, preventing the proliferation of oral pathogens, and inhibiting bacterial adhesion to surfaces. Investigations examining the effectiveness and safety of plant-based medicines have also been presented, including those conducted over the past decade.

## 1. Introduction

**Figure 1:**
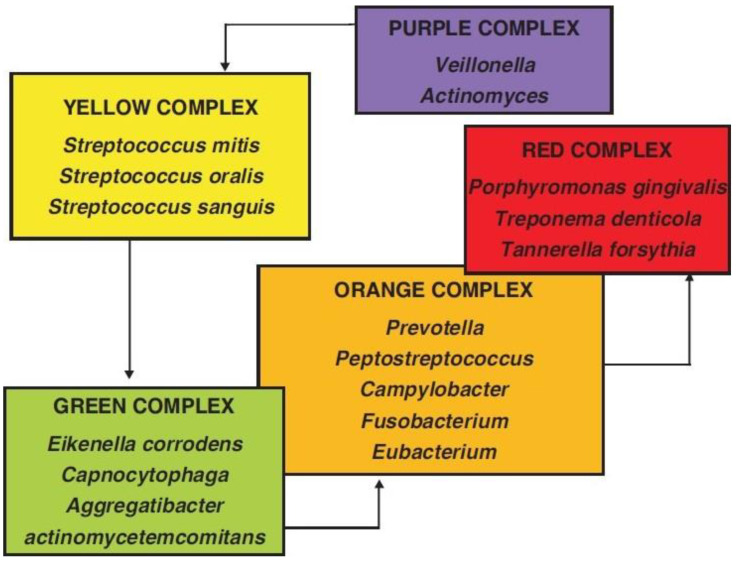
Microbial complexes involved in the progression and development of periodontal diseases [14].

One of the most prevalent diseases influencing the teeth and the supporting tissues, such as the bone, periodontal ligaments (PDL), and cementum, is periodontitis [1]. The word “periodontitis” originates from the Ancient Greek words περί (perí means “around”) + ὀδoύς (odoús means “a tooth”) + -itis. When left untreated, periodontitis spreads from the gingival inflammatory response to the deeper tissues, changing the bone’s homeostasis and destroying the connective tissue attachment, resulting in the loss of alveolar bone, ultimately leading to tooth loss [2]. In adult populations, the prevalence of periodontal disease, particularly in its mild to moderate forms, is significant [3]. In contrast, the incidence of its severe form increases with age, particularly between the third and fourth decades of life [4]. Several factors contribute to periodontal disease [5]. The bacterial biofilm that develops on dental surfaces and its byproducts have been recognized as the primary cause of periodontitis [6,7,8,9]. By demolishing the attachments surrounding the tooth, the toxins released by periodontal pathogens have a crucial impact on the onset of periodontal disease [10]. Periodontal pathogens classified as the “red complex” are most frequently associated with the commencement and development of periodontal diseases among all the bacterial complexes found in biofilm. *T. denticola*, *P. gingivalis*, and *T. forsythia* are the three bacterial species that make up the red complex. The coexistence and elevated levels of all the pathogens in the red complex have been identified in stage III and IV periodontitis [11,12]. On the other hand, stage IV periodontitis of the molar incisor and some stage III and IV periodontitis are commonly associated with *Aggregatibacter actinomycetemcomitans* (*A. a*) [12,13] (Figure 1).

**Figure 2:**
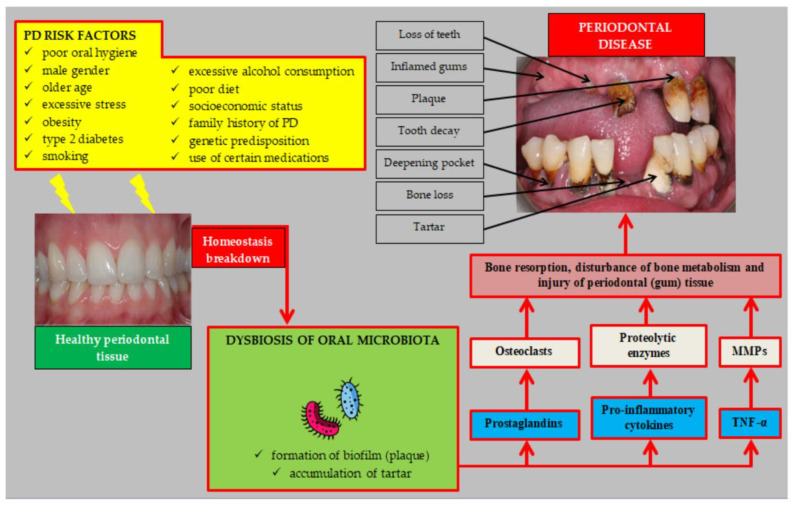
Personal, social, systemic, and local risk factors associated with oral dysbiosis lead to periodontal disease development and progression through activating pathogenic pathways [19].

Along with local factors, including plaque and calculus, the patient’s systemic health, socioeconomic status, lifestyle choices, age, gender, ethnicity, genetics, environmental factors, and host response also affect how the disease progresses [5]. Metabolic syndrome, smoking, diabetes, and obesity are additional significant contributors (Figure 2) [15,16]. Periodontopathogens have detrimental effects on patients’ overall health, in addition to their damaging effects on the periodontium [17]. Leaving periodontal disease untreated may predispose the patient to various systemic diseases. Cardiovascular disease, diabetes, insulin resistance, oral and colon cancer, digestive disorders, adverse pregnancy outcomes, respiratory tract infections, pneumonia, and Alzheimer’s disease are among these conditions [18].

Periodontal treatment seeks to create a root surface that is “biologically acceptable” by eliminating the etiological bacteria and their metabolites [20]. Mechanical debridement is the primary method for treating and preventing periodontal disease, which entails scaling and root planning (SRP). Additionally, chemical plaque control procedures may be used as adjuvants to sustain long-term findings [21]. While surgical periodontal therapy is required in cases of progressive disease, non-surgical approaches can be practical in mild–moderate periodontitis [22]. Irrigating solutions, long-term drug delivery mechanisms, various drug delivery techniques, and mouthwashes are frequently employed as alternative solutions to non-surgical debridement in managing periodontitis [22,23]. In clinical interventions, the most commonly used adjunctive therapies for periodontitis cases are tetracycline, azithromycin, amoxicillin, and metronidazole [24,25,26]. The exponential increase in multidrug-resistant (MDR) bacteria to current antibiotics is a significant concern because it is the leading cause of treatment failure [27]. Therefore, it is crucial to develop antimicrobial medications that stop the emergence of drug resistance and improve the outcomes of treating infectious diseases.

Since time immemorial, plants and their extracts have been employed to achieve therapeutic objectives [21]. When it comes to preventing the emergence of antibiotic resistance in bacterial pathogens, these compounds have shown encouraging results [28]. Herbal medications are suitable substitutes for synthetic medicines in preventing and treating periodontal diseases due to their considerable natural action, broader biological activity, substantial safeness, and lower price [29,30,31]. A large class of chemical compounds discovered naturally in plants are referred to as plant-derived chemicals. These substances are widely present, and their anti-inflammatory, antibacterial, and antioxidant effects have proven advantageous [32]. Antibacterial compounds are widely distributed among plant species [33], and ethnobotanical knowledge can aid in the identification of plant extracts for developing new antibacterials [34,35]. In addition, older antibiotics can be increased in potency to restore their clinical application through the adjuvant effects of herbal extracts added to them, thus preventing the emergence of resistance [36].

Herbal alternatives are an untapped source of potential compounds beneficial in treating many human ailments, such as periodontal diseases, and benefiting overall health. As dietary supplements, herbal products are increasingly used to treat or prevent common diseases [37]. However, regarding the clinical use of these compounds in periodontics, investigation in the field of herbal science is still in its infant stages. The goal of this study was to summarize the research on medicinal plants that has been conducted to support their application as traditional medicine for the management of periodontitis.

## 2. Methods

The Scopus and PubMed/MEDLINE databases and Google Scholar were thoroughly searched. Two reviewers (S.A.M. and H.T.) searched the databases independently. A preliminary investigation was performed of medical botany to list all the plant-based compounds studied in periodontal diseases. The search keywords then comprised a list of medicinal herbs included in the present study combined with the terms “periodontitis” and “periodontal diseases”. The search protocol was conducted separately for each herbal compound using its related keywords. The keywords were searched in English-published papers in journals up to December 2022. To improve the search’s accuracy, the reference lists of the listed articles were manually searched as well.

After screening the retrieved papers, the eligible articles regarding the study’s subject were included. Clinical, in vitro, and in vivo studies published within the last 30 years that investigated any relationship between periodontal diseases and medicinal herbs were included. Non-English papers, posters, abstracts, and studies with inadequate data were all excluded. Two reviewers (H.T. and S.A.M.) then performed the data extraction, extracting the necessary data and recording it on a standardized Excel datasheet. For each plant species, data were compiled on its family, genus, species, parts utilized for medicine, and applications. The type of study, studied samples, methodology, and findings were all included in the extraction form.

## 3. Periodontitis

In the past, periodontal diseases were classified as aggressive or chronic [38]. In a new classification scheme, periodontitis is grouped under one category (“periodontitis”). A multidimensional grading and staging system further characterizes periodontitis. The stages determine both the complexity of the disease management and the severity of the disease at presentation. At the same time, the grades provide additional information on biological characteristics, such as an evaluation of the periodontitis progression rates, an evaluation of the risk of further advancement, and an assessment of the likelihood of poor treatment outcomes and adverse health effects associated with periodontitis [39].

Adaptive and innate immunity are involved in periodontitis. The inflammatory response has four consecutive phases: (1) silence, in which the first proinflammatory mediators are synthesized and released; (2) vascular, where the vascular wall increases in permeability and dilation; (3) cellular, during which inflammatory cells infiltrate the injury site; and (4) inflammatory responses subside [40].

**Figure 3:**
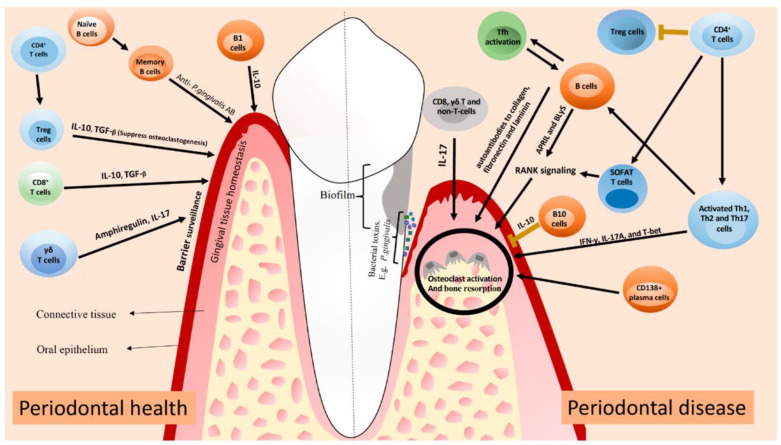
An overview of how the mentioned T and B cells can affect periodontal health. Treg and CD8+ T cells produce IL-10 and TGF-β to maintain periodontal health. To maintain periodontal health, T cells produce amphiregulin and IL-17. Antibodies produced by B cells limit periodontal inflammation. Pro-inflammatory cytokines are released by activated Th1, Th2, and Th17 cells during periodontal disease. A combination of T and B cells produce RANKL, which activates osteoclasts. By clonally activating B cells, Tfh cells can cause local tissue destruction by producing autoantibodies against collagen, fibronectin, and laminin. A lack of Tregs or impaired function probably causes periodontitis. Other cells can also activate osteoclasts by producing IL-17 [45].

In the periodontium, neutrophils, antigen-presenting cells, and T and B lymphocytes form complex networks that interact with the humoral systems, including the complement system, which initiate immune and inflammatory responses [41]. The complement system has many other functions besides tagging and eliminating microorganisms. By synergizing with the Toll-like receptors on innate leukocytes, the complement system can increase immune and inflammatory responses and regulate B and T cell differentiation [42]. Several protein interactions occur in periodontitis, which is responsible for inflammation-induced bone loss. These proteins include the decoy receptor (osteoprotegerin), the functional receptor (RANK), and the receptor activator of the nuclear factor-κB ligand (RANKL). When the periodontium becomes inflamed, the activated lymphocytes (T and B) and osteoblasts produce RANKL [43]. Pre-osteoclasts mature and activate by contacting the RANK receptors on their cell membrane with RANKL. However, osteoprotegerin, a decoy receptor, antagonizes this binding process [44]. Figure 3 discloses the role of immune cells in a healthy periodontium versus periodontitis.

The inflammatory response should be terminated before it becomes chronic and adversely affects the individual. It is crucial to coordinate a series of steps to treat inflammation effectively, as chronic inflammation contributes to many chronic conditions, including periodontitis [46]. In addition to inhibiting neutrophil recruitment, tissue phagocytes clear apoptotic neutrophils via efferocytosis and initiate tissue repair. These processes involve downregulating proinflammatory mediators and upregulating regulatory or pre-resolution mediators [47].

To restore gingival health while protecting the residual periodontal tissues, periodontal therapy minimizes local factors and the bacterial load and corrects behavioral variables such as tobacco use and smoking cessation [48]. The non-surgical treatments for periodontitis include SRP, mouthwashes, dentifrices, and locally and systematically administered antimicrobial medications [49]. Combining mechanical root debridement with the patient’s oral hygiene practices prevents bacterial recolonization and the production of supragingival biofilms [50].

In addition to SRP, chemoplaque management techniques can be used as adjuvants in treating periodontal disease [21]. Patients with periodontitis have been shown to have improved outcomes with SRP and adjuvant antimicrobial agents [51]. The important issue is antimicrobial resistance [52]. Research on dental biofilms has found that antibiotic resistance increases in biofilms when exposed to clindamycin, doxycycline, metronidazole, and moxifloxacin. Herbal medications are recommended as alternatives to synthetic agents because of their natural action, high safety, and lower cost [29,30,31].

## 4. Plant-Based Antimicrobials against Periodontitis

Traditional therapies for various human diseases have used medicinal plants for centuries and in many regions worldwide [53]. Traditional medicines are used for health care by approximately 80% of people in developing nations [54]. Numerous biologically active substances have been developed into new lead chemicals for pharmaceuticals employing natural substances formulated from therapeutic herbs, which have been shown to be rich sources of these substances. There is a lot of potential for identifying new bioactive compounds because roughly 1% of the approximately 500,000 plant species worldwide have undergone phytochemical research [55]. Many cases of oral infections and diseases have been reported to have been treated with traditional plants and natural products [55]. In particular, phytochemicals and extracts from traditional medicinal plants have been shown to reduce dental plaque deposition, inhibit the proliferation of oral pathogenic organisms, and impact their adhesion to dental surfaces, alleviating the consequences of oral diseases [55].

### 4.1. Acacia arabica (Babul)

A commonly used chewing stick in India is *Acacia arabica*, known as “Babul” or “Kikar” datun. Many societies use *Acacia arabica* gum to maintain oral hygiene [56]. The main ingredient is arabica, a complex blend of Arabic acid’s calcium, magnesium, and potassium salts. In addition, tannins, cyanogenic glycosides, oxidases, peroxidases, and pectinases with antibacterial properties are present [57]. *Acacia arabica*’s antibacterial and antiprotease abilities have been established in vitro [58]. Clinical studies comparing *Acacia arabica* gum to CHX have demonstrated its comparable effectiveness in preventing plaque, lowering bacteria counts, and treating gingivitis without any of CHX’s side effects [59,60]. As a result, long-term *Acacia arabica* use is advised.

### 4.2. Acacia nilotica

The tree *Acacia nilotica*, also known in Sudanese folk medicine as “Garad or Sunt”, is found in this country’s central and northern regions. Tannin [61], gallic acid, catechin, epigallocatechin-7-gallate, catechin derivatives [62,63], ellagic acid, kaempferol, and quercetin [64] have all been found in the leaves and bark of *A. nilotica*. Additionally, numerous investigations have demonstrated that it possesses a variety of pharmacological effects, such as anti-HIV-1 protease [65], antibacterial [66], antioxidant, anticarcinogenic [67], and anti-inflammatory characteristics [68]. According to evidence, *A. nilotica* bark has antibacterial potential and inhibitory activity. Moreover, it can be utilized as an adjuvant antioxidant in mouthwashes and to develop future treatment options for periodontal diseases [69].

### 4.3. Allium sativum (Garlic)

**Figure 4:**
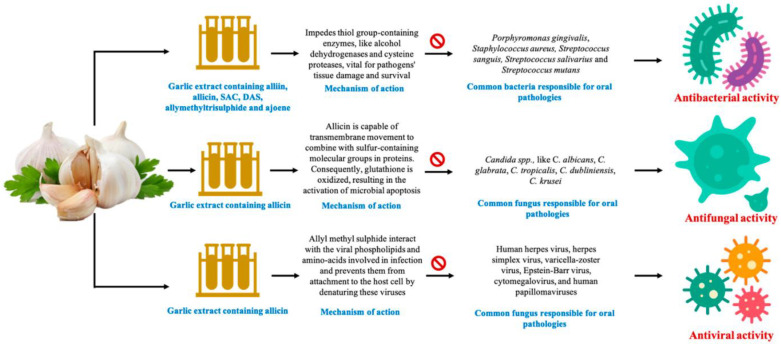
Different mechanisms of action through which garlic extract’s compounds assert antibacterial, antifungal, and antiviral effects [78].

As a medicine, garlic (*Allium sativum*) has been recognized for centuries as having antibacterial, antifungal, and antiviral properties [70,71]. *Allium sativum* is traditionally used in treating infection, diabetes, and cardiac disease. Fresh raw garlic are composed mainly of water (66%), carbohydrates (27%), proteins (2.5%), amino acids (1.3%), fiber (1.6%), phenols, and trace minerals (2.4%) [72]. Garlic extract (GE) may benefit health because of its phytochemicals, including alliin, methiin, and sodium acetate [73]. Alliinase converts garlic alliin into allicin, an antibacterial compound that shows promise for treating periodontal disease, dental caries, and oral cancer [74]. Innovative concepts have emerged with fresh discoveries, such as aged garlic extract (AGE), which has been applied as medicine since 3000 BC. Researchers found that AGE lowered patients’ periodontitis levels more effectively than a placebo [75]. It is widely known that garlic can prevent inflammation, attack bacteria, viruses, and fungi, and prevent mutagenesis [76,77]. Numerous oral microbial diseases may be treated with garlic. Several novel garlic-based products, such as gels, gums, toothpaste, and pharmaceutical strips, have been reported as cost-effective and consumer-friendly solutions for improving oral health [78]. Figure 4 demonstrates the antimicrobial effects of GE against oral microorganisms. Allicin takes 1000 times longer than antibiotics to acquire resistance. Alcohol dehydrogenases and cysteine proteases (vital for tissue destruction) are among the thiol-containing enzymes inhibited by allicin’s antibacterial chemical [73]. A study discovered that taking GE orally reduced both the gingival (GI) and the bleeding index (BI), demonstrating that GE can also reduce periodontal conditions [79]. Tannins, flavonoids, and alkaloids are responsible for GE’s antibacterial activities [80]. As a result, periodontal diseases and dental caries can be successfully treated using garlic bulbs. When administered directly, garlic irritates the mucosa and so must be used carefully [81]. DAS, a sulfur-containing amino acid found in AGE, was shown to suppress the development of periodontal bacteria and reduce the *P. gingivalis*-induced inflammatory responses in human gingival fibroblast cells [82]. Taking AGE tablets helped prevent and enhance periodontal diseases in the long term [83]. According to studies, gingival inflammation and bleeding are reduced when AGE is consumed regularly for at least four months [79]. In recent investigations, garlic has been discovered to have anti-proteolytic properties against *P. gingivalis* protease, as evidenced by AGE’s intense bacteriostatic activity against *P. gingivalis* and gelatin liquefaction after 250 μL/mL dose administration [84]. In 200 individuals with good health, the effectiveness and effects of AGE on periodontitis were examined. Compared to the baseline value (1.50 ± 0.46), the mean PD for AGE after ten months was 1.06 ± 0.49, showing that AGE might help prevent or decrease periodontitis. Garlic’s bioactive components may suppress oral infections and some proteases, which may benefit patients with periodontitis [83].

### 4.4. Aloe barbadensis Miller (Aloe Vera)

**Figure 5:**
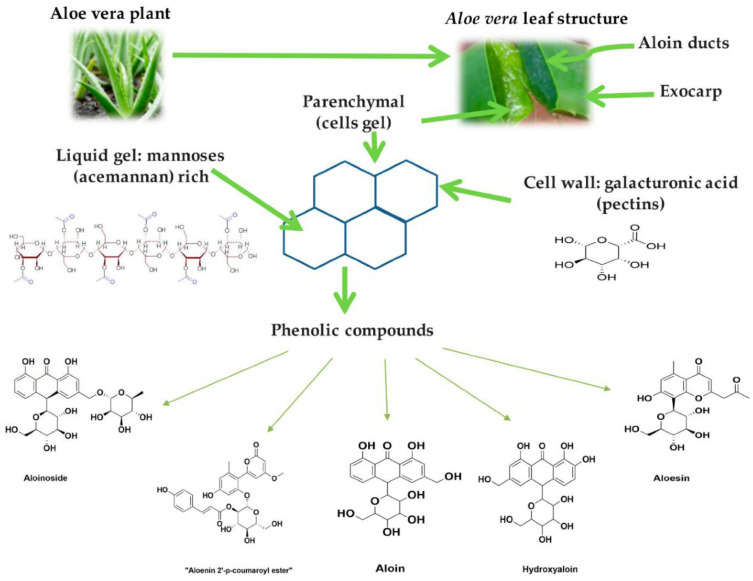
The main phenolic compounds of the Aloe vera plant and their chemical structures [102].

Therapeutic uses of Aloe vera date back thousands of years. In addition to treating bruising, X-ray burns, skin infections, hemorrhoids, sinusitis, gastrointestinal pain [85], and insect bites, this medicinal plant is also an anti-helminthic, somatic, and anti-arthritic [86,87]. Among the 75 constituents of Aloe vera are minerals, enzymes, sugars, anthraquinone, and salicylic acid [88]. Approximately 99.5% of Aloe vera leaves contain water and 0.0013% protein [87]. Figure 5 shows the primary constituents of an Aloe vera plant. Aloe vera gel has been shown to have pharmacokinetic activities that include anti-inflammatory, antibacterial, antioxidant, immune-stimulating, and hypoglycemic effects [89,90]. Aloe vera has antimicrobial effects on *Streptococcus pyogenes* and *Enterococcus faecalis* [91,92]. Isorabaichromone, feruoylaloesin, and p-coumaryl aloesin, three aloesin derivatives, have demonstrated the potential to scavenge radicals and superoxide anions [93,94]. It is perfect for treating gingivitis and periodontitis due to having an anti-inflammatory compound (C-glucosyl chromone), inhibiting the COX pathway, reducing PGE2, and breaking down the bradykinin inflammatory agent responsible for pain generation [93,95,96]. Edema, bleeding, and irritation of the gingival tissues are reduced by using it. It is beneficial in deep pockets where routine cleansing is challenging, and its antifungal properties also help treat denture stomatitis, aphthous ulcers, and angular cheilitis [97]. Using it after extractions is a powerful healer [98]. In root canal therapy, it has been used as a sedative dressing and file lubricant [99]. Many studies have been performed to determine if Aloe vera effectively cures gingivitis. In a double-blinded trial, 120 volunteers were requested to skip two weeks of tooth brushing. After being separated into three groups, 100% Aloe vera, distilled water as a placebo, and 0.2% CHX were given to the patients. The Aloe vera mouthwash was beneficial in lowering plaque and gingivitis, although when compared to CHX, its effects were not as noticeable [100]. Another study investigated how toothpaste with a high Aloe vera content affected the remission of plaque and gingivitis. The subjects were observed over three six-month periods using either Aloe vera toothpaste or a regular one. After the clinical experiment, the plaque and gingivitis indices decreased by roughly 20%, with no significant difference between the two study groups. Individuals motivated to improve their dental hygiene practices did not experience extra anti-plaque or -gingivitis when using an Aloe vera toothpaste [101]. Using Aloe vera as a medication in periodontal pockets was highlighted in a study performed by Geetha et al. [85].

**Figure 6:**
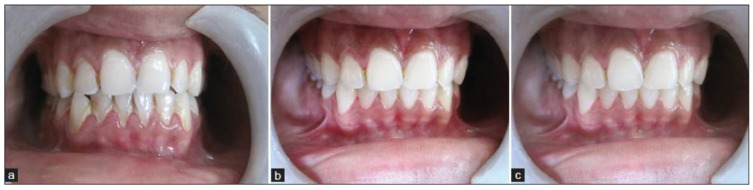
Results of a study on beneficial anti-inflammatory effects of Aloe vera + scaling treatment [(**a**) baseline; (**b**) one-month post-op; and (**c**) three-month post-op] to reduce plaque-induced gingivitis [103].

In Ajmera et al.’s study, Aloe vera mouthwash reduced plaque-induced gingivitis inflammation. Three months of Aloe vera mouthwash (BID) were administered to Group 1. Group 2 was scaled only. Group 3 received Aloe vera mouthwash and scaling. In contrast to the other two groups, Aloe vera mouthwash and scaling were more effective in reducing gingival inflammation. Consequently, Aloe vera was found to be anti-inflammatory, and combined with mechanical therapy, it helped treat plaque-induced gingivitis (Figure 6) [103].

### 4.5. Amphipterygium adstringens

A Mexican endemic species of the *Julianaceae* family called “Cuachalalate” is *Amphipterygium adstringens* [104]. Anacardic acid [105], which has antioxidant, anti-inflammatory [106], anticancer [107], antiulcer, and antibacterial effects [105,108], is the primary ingredient responsible for the plant’s capabilities, according to recent studies.

### 4.6. Azadirachta indica [109]

A member of the *Meliaceae* family of mahogany trees, the neem tree (*Azadirachta indica*) is an evergreen that grows naturally in India and Myanmar’s subcontinent [110,111]. It has been found that extracts from various portions of this tree contain a variety of polyphenols, such as tannins, lignins, and flavonoids, that are potent antioxidants, antibacterials, anti-inflammatory agents, and immunomodulators [110,111,112,113,114,115,116,117,118,119,120]. The chewing sticks produced from twigs of the tree may play a role in oral care due to their mechanical cleansing properties, stimulation of saliva secretion, and antibacterial and antioxidant properties [121]. Aqueous preparations of neem have shown antimicrobial properties by reducing the surface adhesion of specific bacteria, destructing bacterial cell membranes, and inhibiting bacterial growth [115,116,122,123]. The plaque buildup and bacterial counts were significantly reduced after oral neem extract therapy [124]. With antioxidant properties, a neem extract may reduce the oxidative stress associated with periodontal disease and have anti-inflammatory potential [110,111,125]. Neem may have anti-inflammatory properties by suppressing prostaglandin E and 5 HT, reducing inflammation [123].

### 4.7. Berberis vulgaris

Extracts of *Berberis vulgaris* (*Berberidaceae* family) root exhibit antibacterial activity against periodontal bacteria due to berberine, the principal active ingredient. The growth of *P. gingivalis* and *A. a* has been shown by researchers to be inhibited by berberine [126,127,128]. *P. intermedia*, *Actinomyces naeslundii*, and *Prevotella nigrescens* do not grow due to the bacteriostatic properties of berberine [126,129]. The microbiological activity of a dental gel containing barberry root extract was investigated [130]. It was demonstrated that the protoberberine alkaloids had a synergistic antibacterial action, which can be used to explain why *P. gingivalis* growth was suppressed at 0.015 mg/g [130]. The plaque index (PI) was found to have decreased in a trial of the efficacy of a dental gel containing 1% berberine. Comparatively, applying a 5% gel reduced the growth of invading bacteria [129].

### 4.8. Camellia sinensis (Green Tea)

*Camellia sinensis* belongs to the *Theaceae* family and has small perennial shrubs widely used to produce green and black teas [131]. Its beneficial properties are attributed to green tea’s polyphenol components (catechins). Epicatechin-3-gallate and epigallocatechin-3 gallate are the two significant catechins. Compared to black tea, green tea contains higher polyphenols (30–40% vs. 3–10%), with enhanced antioxidant capacity and strong anti-inflammation, antibacterial, antiviral, antimutagenic, and anti-aging activities [132,133,134]. Inflammation and periodontitis are positively affected by green tea. Thus, research supports green tea as a curative and preventive agent for periodontal disease [135].

### 4.9. Cinnamomum zeylanicum (Ceylon Cinnamon)

Cinnamon has been utilized as a culinary herb in traditional medicine. Cinnamon has been researched in pregnancy, diabetes management [136], and gynecological disorders [137]. It has anti-inflammatory, cardioprotective, antioxidative, and antibacterial activities and anti-inflammatory capabilities [138]. Cinnamon refers to a collection of around 250 evergreen trees belonging to the *Lauraceae* family [139]. Several species have been studied, including those linked to oral medicine. *Cinnamomum verum* and *Cinnamomum zeylanicum* are two of the most studied cinnamon types. *Cassia cinnamon*, often known as Chinese cinnamon or *Cinnamomum aromaticum*, is a well-studied spice. *Cinnamomum burmannii* and *Cinnamomum loureiroi* are two more major cinnamon species [138,140]. The EO of *Cinnamomum* bark (CBEO) contains many aromatic compounds and high concentrations of cinnamaldehyde and eugenol. CBEO and cinnamaldehyde have antibacterial, antifungal, anti-inflammatory, and anticancer properties [141,142,143]. According to Wang et al., the cinnamaldehyde in *C. zeylanicum* bark EO works against *P. gingivalis* [144]. According to reports, cinnamaldehyde is responsible for CBEO’s antibacterial effect [144]. The relative mechanism of cinnamaldehyde was uncovered by examining the cell microstructure, membrane integrity, and membrane properties [145]. CBEO and cinnamaldehyde may irreversibly damage bacterial membranes, thus compromising membrane integrity. The metabolism will err when the cell membrane depolarizes, and the bacteria will die [144]. As determined by propidium iodide uptake tests, the CBEO and cinnamaldehyde treatments interrupted the integrity of the bacterial membranes. The confocal microscopy analysis of *P. gingivalis* detected PI incorporation, indicating a cell membrane disruption [144]. Microorganisms can be killed by this principal mechanism, which is known as membrane damage [146]. *P. gingivalis* may therefore be susceptible to membrane permeabilization caused by CBEO and cinnamaldehyde.

Eugenol, a compound more commonly associated with clove, is also a potent component of cinnamon EO [147]. Due to its powerful antibacterial properties and abundance in cinnamon EO and extracts, it has been demonstrated to be beneficial to periodontal health. In addition to having antibacterial properties, eugenol has multiple mechanisms of action [148] through the destruction of the cell membrane in a dose-dependent fashion and reducing the presence and formation of the biofilm [148]. Cinnamaldehyde has also been declared safe and non-toxic by the FDA. Cinnamaldehyde can be absorbed quickly by the gastrointestinal system [149]. Nearly no residues are left when the body removes the metabolites [150].

### 4.10. Citrus sinensis

In the *Rutaceae* family, oranges are classified as *Citrus sinensis*, a sweet and juicy fruit. Orange trees are often grown in tropical and subtropical climates because of their medicinal properties and sweet juice. Aside from preventing and treating vitamin deficiency, colds, flu, and scurvy, it also fights bacterial and viral infections [151]. Antibacterial properties have also been reported for orange peel [152]. Dubey et al. demonstrated the robust antibacterial properties of orange peel extracts against different bacteria using the disk diffusion method [153]. The effectiveness of orange peel extract against *Klebsiella pneumoniae* has been demonstrated by Jabuk et al. [154]. Numerous studies [109,151,152,155] have revealed that *Citrus sinensis* can also treat periodontal disease (Table 1 and Table 2).

### 4.11. Coffea canephora (Coffee)

The primary phenolic acid in coffee, chlorogenic acid, acts on human health due to its various effects, such as its antioxidant, anti-inflammatory, and antibacterial properties [199,200,201,202,203]. The safety of chlorogenic acid in rats and dogs is well documented, although there are no reports about humans, except for a potential allergic reaction [204]. Green coffee extract’s chlorogenic acid reduced the quantity of the oral bacteria *S. mutans* in a clinical experiment [205]. There is evidence that coffee extract is antibacterial and inhibits the activity of proteases produced by periodontitis-causing organisms, such as *P. gingivalis* [206].

### 4.12. Copaifera pubiflora

*Copaifera pubiflora* (*Fabaceae-Caesalpinioideae*) plants are indigenous to tropical areas of Western Africa and Latin America. Copaiba is the common name given to these plants in Brazil. The plants produce oléoresin as a byproduct of their secondary metabolism to protect themselves against animals, fungi, and bacteria [207,208,209,210,211,212,213,214,215]. Numerous studies have suggested that *Copaifera* can act against the bacteria responsible for endodontic infections and dental caries [208,209,210,213]. The antibacterial and antivirulence activity was tested against *P. gingivalis* and *A. a* by Abrão et al. These compounds were helpful as antimicrobials against periodontal pathogens [216].

### 4.13. Coptidis rhizoma

The medicinal plant *Coptidis rhizoma* (CR) is a member of the *Ranunculaceae* family [126]. Current investigations indicate that a chemical called berberine (BBR) is the principal active ingredient in CR extract [126]. CR and BBR have various antimicrobial, anti-inflammatory, antifungal, antidiarrheic, and other functions [126,217,218]. BBR therapy exerts anti-inflammatory action by inhibiting MMP-2 and MMP-9 activation, thus reducing periodontal tissue damage in periodontitis [219]. By reducing the synthesis of monocyte chemoattractant protein-1 from affected PDL cells, BBR might reduce leucocyte infiltration into the periodontium [220]. A rat periodontitis model was treated with oral BBR therapy for seven weeks, significantly reducing alveolar bone resorption [220,221]. BBR effectively reduced local and systemic inflammation in a periodontitis rat model by lowering TNF-α and IL-17 production and the number of IL-17A+ cells in the alveolar bone [222]. An in vivo experiment by Gu and colleagues on rats with ligation-induced periodontitis showed that BBR prevents alveolar bone loss caused by inflammation [223]. It was discovered that the enzyme PCSK9, which stimulates inflammatory reactions in the body, was a novel target of BBR’s anti-inflammatory effect. *P. gingivalis*-induced periodontitis was significantly reduced by BBR therapy by lowering PCSK9 production, which was also associated with the suppression of inflammatory responses [224]. Activated T cells in periodontitis produce the cytokine RANKL, which leads to osteoclastic activity and the destruction of alveolar bone [43]. The formation of RANKL is reduced by BBR, preventing bone loss in periodontitis [224].

### 4.14. Curcuma longa (Turmeric)

**Figure 7:**
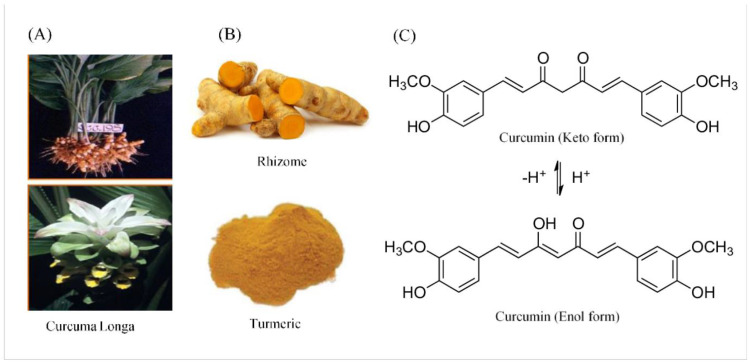
The botanical source of turmeric (**A**). Powdered curcumin (**B**). Curcumin in enol and keto forms (**C**) [235].

Southeast Asia is home to the ginger family member *Curcuma longa*. Curcumin’s capacity to inhibit LOX and COX activity in people is the basis for its well-documented anti-inflammatory action [225,226]. By controlling inflammatory pathways and activating transcription factors such as activator protein-1, mitogen-activated protein kinase (MAP Kinas), and NF-κB of activated B cells, curcumin has anti-inflammatory actions [176,227]. Additionally, evidence suggests that curcumin exerts healing effects on periodontal conditions and gingival inflammation by efficiently inhibiting the activation of inflammatory mediators [176,228]. Turmeric has been discovered to be a potent anti-inflammatory when used as mouthwash [176]. Curcumin combined with SRP has been shown to boost periodontal parameters. The periodontal indices are also better when curcumin is compared to ornidazole gel [175,176,228,229,230,231]. Kandwal et al. found no appreciable differences in the plaque or GI between CHX and curcumin gels [232]. A possible treatment for periodontitis is being explored by researchers thanks to curcumin’s ability to block the effects of Toll-like receptors [233]. Figure 7 demonstrates the chemical structures of various forms of curcumin. To determine its effects on alveolar bone loss, curcumin was studied in a meta-analysis study. The best results regarding the bone volume fraction and millimeters were obtained with chemically modified curcumin [234].

### 4.15. Cymbopogon citratus (Lemongrass)

The medicinal plant *Cymbopogon citratus* is used to cure various illnesses [236]. According to reports, its chemical constituents, including phenol and flavonoids, exhibit antioxidant, anti-inflammatory, and antimutagenic properties [237]. Lemongrass EO can prevent bacterial growth at a concentration of ≤2% [238]. Hongkhunthian et al. found that it had antibacterial properties against periodontal pathogens, which formerly were resistant to tetracycline [239]. Gingivitis can be effectively treated with lemongrass EO mouthwash as a non-surgical adjunct to standard remedies [31,240]. Mucoadhesive polymer-based semi-solid formulations have been proposed to enhance contact quality and lengthen the dosage form’s persistence in the deep periodontal pocket, where conventional mouthwashes have difficulty penetrating [241]. The antioxidant properties of these EOs may account for their anti-clastogenic effects [242].

### 4.16. Eucalyptus globulus

The fever tree, or *Eucalyptus globulus*, belongs to the *Myrtaceae* family. Eucalyptus EOs have widespread use throughout the globe, are considered safe and non-toxic, and are approved for use as a food flavoring ingredient [243]. Eucalyptus leaves’ EOs, flavonoids, and tannins are considered responsible for their antioxidant, larvicidal, anthelmintic, antibacterial, and fumigant properties [244]. Oral pathogens such as *streptococci* are frequent among the oral bacteria that antibacterial ethanol extracts from the leaves of *E. globulus* target [245]. Additionally, the extracellular glucosyltransferase from these bacteria is inhibited by the extracts from producing insoluble glucan [246]. Ethanol extracts of *E. globulus* leaves also showed antibacterial action against two periodontal bacteria: *P. gingivalis* and *P. intermedia*. *P. gingivalis*, a periodontopathic bacterium, was significantly suppressed at modest concentrations [247]. A study demonstrated statistically significant positive effects on gingivitis outcome indicators with chewing gum containing 0.6% extract of *E. globulus* leaves [247].

### 4.17. Garcinia mangostana

The *Guttiferae* family includes *Garcinia mangostana*, more commonly referred to as mangosteen or the “queen of fruits”. It is an evergreen tree that originated in Southeast Asia [177]. The pericarp of this plant contains chrysanthemum, garcinone, sesquiterpenoids, gartanin, fructose, sucrose, tannins, and other beneficial chemicals [177]. Among its many properties, *mangostana* has antibacterial, antioxidative, anticancer, antiproliferative, and pro-apoptotic effects, and it exhibits aromatase inhibition [248,249]. Mangosteen is high in xanthones, a polyphenol molecule with significant biological properties. It is also high in flavonoids and anthocyanins [250,251]. Additionally, regular *mangostana* may be beneficial in preventing numerous pathological illnesses caused by oxidative stress and inflammation [252]. One study reduced the growth of *P. gingivalis* using an 80% ethanolic extract of *mangostana* pericarp gel at an MIC of 3.91 g/mL [253]. Researchers observed significant improvements in the periodontal parameters of patients with chronic periodontitis after locally applying 4% *mangostana* gel in their periodontal pockets [177]. According to a recent study, combining mangosteen and propolis extract significantly reduced the production of IL-6, IL-8, and PGE2 in immortalized human cells treated with *P. gingivalis* lipopolysaccharides. Furthermore, it stimulated human osteoblast-like cells to produce the highest bone-forming activity [254].

### 4.18. Glycyrrhiza glabra and Glycyrrhiza uralensis (Chinese Licorice)

Chinese and Ayurvedic medicine have used licorice root for centuries. The *Glycyrrhiza* species native to Europe and Asia contain licorice, a sweet, moist, alleviating plant [255]. Licoricidin and licorisoflavan A, the primary isoflavans from Chinese licorice (*Glycyrrhiza uralensis*), inhibited the proliferation of *P. gingivalis*, the generation of volatile sulfur compounds (VSCs), and the protease activity resulting in halitosis [256]. Research suggests that using licorice can prevent gingivitis and promote oral health. After pre-treatment of *P. gingivalis* with licorice root polysaccharides, Witttschier et al. discovered that these polysaccharides might inhibit bacterial binding from host cells. According to the study, polysaccharides from *G. glabra* inhibit bacterial adhesion [257]. When macrophages are activated with *A. a* and *P. gingivalis*, licorice extract demonstrates powerful anti-inflammatory activities by suppressing the periodontopathogen LPS-induced IL-1β, IL-6, IL-8, and TNF-α responses [258]. MMPs and inflammatory cytokines are well inhibited by licoricidin and licorisoflavan A, according to La et al.; thus, they can treat periodontitis [259]. The host immunological response and biofilm development by *P. gingivalis* are inhibited by licochalcone A [260]. Recently, the efficiency of licorice extract in inhibiting MMP production by host cells in patients with chronic periodontitis was established [261].

### 4.19. Hibiscus sabdariffa

In English, *Hibiscus sabdariffa*, often called roselle or red sorrel, belongs to the *Malvaceae* family and is an extensively cultivated plant in Southeast Asia and Central and West Africa. Tropical or subtropical climates favor its growth [262,263]. Many secondary metabolites are present in the calyx of roselle, such as flavonoids, alkaloids, saponins, and hibiscetin [264,265]. Roselle also contains delphinidin-3-sambubioside, which inhibits osteoclastogenesis by decreasing inflammatory mediator synthesis. Due to its anti-inflammatory and antibacterial properties, it may be used to address alveolar bone loss [266,267,268,269]. Roselle’s antibacterial potential may help prevent plaque development, leading to the prevention of further bone destruction [270,271,272,273]. Its anti-inflammatory properties have also been shown in previous research on its extract [274,275,276].

### 4.20. Inula viscosa (False Yellowhead)

The *Asteraceae* family includes *I. viscosa* (*Dittrichia viscosa*), which grows mainly in the Mediterranean region [277]. *I. viscosa* was demonstrated to exhibit minimal bactericidal concentrations (MBC) of 0.15 mg/mL against obligate anaerobes such as *P. gingivalis*, with minimum inhibitory concentrations (MIC) ranging from 0.07 mg/mL (*P. gingivalis*) to 2.50 mg/mL (*S. sobrinus*) against selected oral bacterial species [278]. In situ, early oral biofilms are not yet known to be susceptible to *I. viscosa*’s antibacterial effect. Scabies and skin irritations such as eczema are treated with *I. viscosa* as a folk medicine plant [279,280]. *I. viscosa* extract was reported to have anticancer, antioxidant, antifungal, antibacterial, and hypoglycemic properties [281]. In addition, *I. viscosa* tea decreased the adhering bacteria in the primary in vivo oral biofilm without harming the salivary pellicle’s ability to protect against acid [282]. *I. viscosa*, based on the research, shows significant potential to preserve oral health, especially when its diverse components come into contact with the oral mucosa.

**Figure 8:**
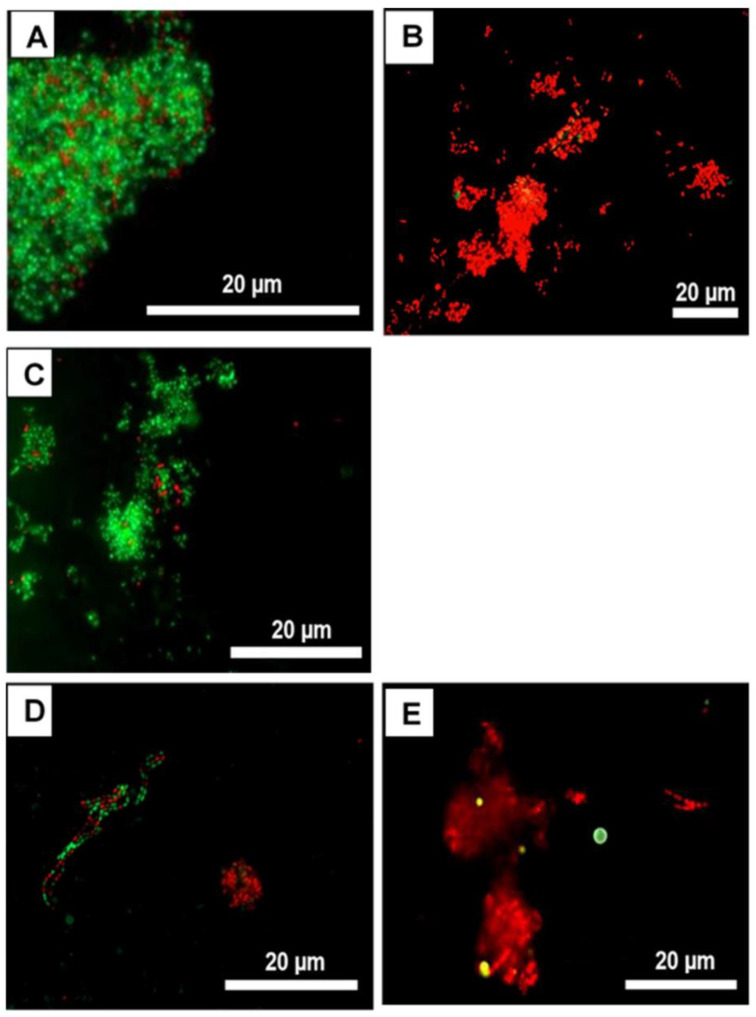
Live/dead bacLight^®^ fluorescent microscopy images. Avital fluoresces are in red, while vital bacteria are in green. (**A**) NaCl treatment as negative control, (**B**) CHX treatment as positive control, and (**C**) DMSO treatment as toxicity control, *I. viscosa* groups in concentrations: 10 mg/mL (**D**) and 30 mg/mL (**E**) [283].

Using *Inula viscosa* extract to inhibit microbial adhesion in the oral cavity, Kurz et al. studied its antimicrobial effect. In this study, bovine enamel samples were attached to individual test splints for each participant. Fluorescence microscopy, colony-forming units (CFU), and vitality testing were used to assess microbiological parameters. Figure 8 displays live/dead samples of oral biofilms after applying *I. viscosa* extract at different concentrations. The untreated control and the DMSO-treated control (Figure 8) exhibited condensed accumulation of viable [39] bacteria. Almost no avital (red) bacteria were detected, and the bacterial arrangements were diverse. CHX and *I. viscosa* extract (Figure 8) significantly affected the oral biofilms. Initially, most adhering bacteria were red (avital) [283].

### 4.21. Juglans regia

One of the most valuable medicinal plants is the walnut tree, or *Juglans regia*, which is beneficial in the therapeutic and cosmetic domains [284]. Various regional names are also used in other civilizations, including Derum, Dandasa, and Sewak. Multiple studies have examined the shells, kernels, seeds, and bark of *Juglans regia*, among other aspects [285]. The bark of *Juglans regia* may be used as a teeth-cleaning agent or a lip colorant in the cosmetic industry [286]. As a fibrous, resinous, and fragrant part, *Juglans regia* bark comes in several forms and sizes [287]. A variety of disorders can be treated with the bark of *Juglans regia*, which has anti-inflammatory, blood purification, anticancer, depurative, diuretic, and antioxidant properties [288]. Its antifungal and antibacterial properties have been proven to exert inhibitory action [289]. *Juglans regia* bark extracts showed broad-spectrum antibacterial efficacy against various pathogens, including Gram-positive and Gram-negative bacteria, in a dose-dependent manner [290]. Several studies showed *Juglans regia*’s antimicrobial activity (Table 1 and Table 2). *Juglans regia* contains terpenoids, alkaloids, steroids, phenols, and flavonoids used in oral hygiene products [290]. A recent study showed that juglone, a bioactive component of *Juglans regia*, inhibits *P. gingivalis* growth and antibiofilm action (*S. sobrinus*, *A. viscosus*, and *S. mutans*). In mice and rats, septa and leaf extracts demonstrated minimal toxicity [155,291]. *Juglans regia* is a good product for enhancing dental and oral health based on its antiplaque activity [291].

**Table 2:** In vitro and in vivo investigations of plant-based antimicrobials in periodontal diseases.

Natural Compound	Study Type	Samples Studied	Methods	Result(s)/Conclusion(s)	Ref./Year
*Acacia nilotica*	In vivo	Albino rabbits with ligature-induced periodontitis	G1: Distilled water G2: Positive control group G3: *A. nilotica* aqueous extract (dosage 300 mg/kg) G4: *A. nilotica* aqueous extract (dosage 500 mg/kg) G5: Amoxicillin (15 mg/kg) [CBC, ESR, serum creatinine, ALT, and AST were measured after 14 days]	*A. nilotica* extract significantly cured periodontitis to a great extent after 14 consecutive days of oral administration.	[292]/2019
*Allium sativum*	In vitro	*P. gingivalis*, *F. nucleatum*, *A. a.*	Gs: An aqueous extract of *Allium sativum* [Disc diffusion technique, microspindle dilution method, and assessment of MIC and MBC were performed]	*Allium sativum* extract at 55.2% *w*/*v* produced inhibition zones of 17.3 ± 1.0, 30.3 ± 1.7, and 21.2 ± 2.3 mm with *A. a*, *F. nucleatum*, and *P. gingivalis*, respectively. MIC of 17.2, 1.1, and 4.3 mg/mL was obtained for *A. a*, *F. nucleatum*, and *P. gingivalis*, respectively. The MBC was 34.4, 1.1, and 8.6 mg/mL, respectively. *Allium sativum* aqueous extract may be a therapeutic alternative for treating periodontal disease based on the results obtained in this study.	[293]/2021
	In vitro	*L. acidophilus*, *S. aureus*, *S. sanguis*, *S. mutans*, *S. salivarius*	Gs: *Allium sativum* bulb [MIC and MBC were measured]	*A. sativum* bulbs are effective in treating periodontitis and dental caries. MBC value ranged from 60 ± 5 to 215 ± 7 mg/mL and MIC value ranged between 20 ± 2 and 120 ± 6 mg/mL.	[80]/2020
	In vitro	*P. gingivalis*	G1: Aqueous garlic extract G2: 0.2% CHX [Groups were compared regarding MIC and MBC]	A significant difference was observed between G1 and G2 (0.29 ± 0.1 μL; *p* < 0.001) regarding the MIC (1.21 ± 0.37 μL) and MBC (1.44 ± 0.67 μL) against *P. gingivalis.* As compared to G1 (20.1 ± 1.4 mm), G2 (27.3 ± 1.8 mm) showed a significantly larger inhibitory zone against *P. gingivalis* (*p* < 0.000). Garlic extracts performed well as antimicrobial agents against *P. gingivalis*; however, they were not superior to CHX as antimicrobial agents.	[294]/2019
*Aloe barbadensis Miller*	In vitro	*C. albicans*, *S. mutans*, *L. acidophilus*, *E. faecalis*, *P. intermedia*, *P. anaerobius*	G1: Aloe vera tooth gel G2: Pepsodent toothpaste G3: Colgate toothpaste [Zone of inhibition was measure]	In preliminary tests, Aloe vera tooth gel and other toothpastes had similar antibacterial effects. *S. mitis* benefited from an enhanced antibacterial impact by Aloe vera tooth gel (*p* = 0.034).	[295]/2009
*Amphipterygium adstringens*	In vitro	*S. mutans*, *P. gingivalis*, *A. a*, *E. coli*, *C. albicans*, *C. dubliniensis*	Gc: 0.12% CHX Gs: A methanolic extract of *A.* *Adstringens* [MIC, MBC, and total growth inhibition were measured]	All microbial strains tested with methane extracts of *A. adstringens* exhibited antimicrobial activity between 0.125 and 63 mg/mL. MIC of *S. mutans* was 0.125 mg/mL, and MBC was 0.31 mg/mL, making it the most sensitive strain. The MIC and MFC of Candida strains were 0.4 and 1.6 mg/mL, respectively. An MIC/MBC of 37 mg/mL was observed for both *P. gingivalis* and *E. coli*. With an inhibitory concentration of 63 mg/mL, *A. a* and *E. coli* also exhibited similar results. An MBC of 2.4 mg/L was found for chlorhexidine.	[296]/2015
*Azadirachta indica*, *Syzygium aroticum*, and *Cinnamomum zeylanicum*	Ex vivo	*Actinobacillus* sp.	Gc: Tetracycline and azithromycin (30 mcg/mL) Gs: Neem, clove, and cinnamon in aqueous and acetone solvents (2%, 4%, 6%, 8%, and 10%) [Zone of inhibition was measured]	*Actinobacillus* sp. were inhibited at 10% concentration by aqueous extracts of clove and neem (24 and 22 mm, respectively). At the same concentration, aqueous cinnamon extracts displayed only a moderate inhibition zone (16 mm). Acetone extracts of neem and clove showed effective inhibition of *Actinobacillus* sp. (20 and 18 mm, respectively) compared with cinnamon, which showed a moderate inhibition zone (14 mm). Neem, clove, and cinnamon extracts could be used as an alternative treatment for chronic periodontitis.	[297]/2020
*Berberis vulgaris*	In vivo	Rats with ligature-induced periodontitis	Gc: Cholisal gel Gs: A dental gel containing barberry extract. [Histopathology and ultrasound dopplerography were performed]	Periodontitis can be effectively treated with a dental gel containing 0.015 mg/g of barberry root extract.	[130]/2020
*Cinnamomum burmanii*	In vitro	An *A. a*. or *E. coli* LPS-stimulated macrophage model	Gs: Cinnamon bark aqueous extract [Cytokine production, binding of LPS cells, and PPAR-γ binding were studied]	IL-6, TNF-α, and IL-8 secretion was reduced by the cinnamon fraction in a dose-dependent manner. A cinnamon fraction may have anti-inflammatory properties by reducing LPS binding to monocytes. A natural PPAR-γ ligand may exist in the cinnamon fraction as well. A cinnamon fraction has been shown to contain anti-inflammatory properties that can be used to treat periodontal disease due to its anti-inflammatory properties.	[298]/2021
*Cinnamomum zeylanicum*	In vitro	*P. gingivalis*	Gs: Different concentrations of cinnamon with oil solvent (10, 50, 100, 250, 500, 750, and 1500 mg/mL) Gc: Amoxicillin, metronidazole, ciprofloxacin, amikacin, and gentamycin [MBC and MIC were evaluated]	Cinnamon at an MIC value of 750 mg/mL inhibited bacteria, while cinnamon at an MIC value of 1500 mg/mL killed them. The antibacterial activity was, however, much weaker than that of common antibiotics (*p* < 0.001). The antimicrobial activity of cinnamon against the pathogen *P. gingivalis* was demonstrated in patients with chronic periodontitis with deep pockets.	[299]/2018
	In vitro	*A. a*, *F. nucleatum*, *P. gingivalis*, *S. salivarius*, *S. mitis*, and *S. mutans*	Gs: EO from cinnamon tree bark + cinnamaldehyde [MIC was measured]	An MIC of 0.21–0.63 mg/mL was observed for cinnamon oil and 0.8–0.15 mg/mL for cinnamaldehyde against the tested bacteria. Changes in cell membranes were observed after two hours of exposure to the oil. Bacterial infections of the oral cavity can be prevented by cinnamon bark oil.	[300]/2013
*Citrus sinensis*	In vitro	*P. gingivalis*	Gs: *Citrus sinensis* [MIC, SI, and IC_50_ were measured]	*Citrus sinensis* exhibited low cytotoxicity and good antibacterial activity. It demonstrated an IC_50_ value of 512 μg/mL.	[155]/2020
*Coffea canephora*	In vitro	*P. gingivalis*	Gs: Coffee extract and chlorogenic acid [The inhibitory effect, protease activity, and viability of *P. gingivalis* were evaluated]	Chlorogenic acid had an MIC of 4 mg/mL and an MBC of 16 mg/mL. When chlorogenic acid is applied above the MIC, the viability of *P. gingivalis* is inhibited for a longer period of time and the activity of the associated protease is significantly reduced. Different roast levels of coffee had no effect on the antibacterial activity of the extract.	[206]/2019
*Coptidis rhizoma*	In vitro	*A. naeslundii A. a*, *P. gingivalis*, *P. nigrescens*, *P. intermedia*	Gs: *C. rhizoma* extract. [MIC and IC_50_ were measured]	MICs of 0.031–0.25 mg/mL inhibited the growth of the mentioned bacteria, while MICs of 0.5–2 mg/mL inhibited the growth of *Lactobacillus* and *Streptococcus*. *C. rhizoma* extract inhibited periodontopathogenic bacteria. Clinical application of these results may be possible for treating periodontal diseases.	[126]/2000
*Curcuma longa*	In vivo	Rats with induced periodontitis	Gs: Curcumin-loaded nanoparticles	The μCT analysis demonstrated significant attenuation of NF-kB activation and p38 MAPK activity resulting from curcumin local administration. Inflammatory bone resorption, osteoclast counts, and inflammation infiltrates were significantly reduced. Experimental periodontal disease was effectively treated with curcumin-loaded nanoparticles.	[301]/2018
	In vivo	Wistar rats with ligature-induced periodontitis	G1: Placebo G2: Resveratrol G3: Curcumin G4: Resveratrol + curcumin [Morphometric analysis of bone loss was performed histologically; TNF-α, IL-4, IFN-γ, and IL-1β were studied]	As compared with the other groups, G1 showed greater bone loss than the other groups based on intergroup comparisons (*p* < 0.05). G2, G3, and G4 did not have different bone-loss values (*p* > 0.05). In G4, IL-1 β levels were lower than in G1 based on the immunoenzymatic assay of gingival tissue (*p* < 0.05). In comparison with G1, G2, and G3, G4 showed higher IL-4 values (*p* < 0.05). The levels concerning IFN-γ were only reduced by G2 (*p* < 0.05). Among the four groups, the TNF-α concentrations did not differ (*p* > 0.05). There was a reduction in alveolar bone loss due to resveratrol and curcumin. It was not found that these agents combined or synergized in any way.	[302]/2017
*Cymbopogon citratus*	In vitro	*S. mutans*, *S. epidermidis*, *Lactobacillus*	Gc: Tetracycline Gs: Lemongrass EO [Inhibition zone measurement]	The minimal inhibitory concentration of lemongrass EO was estimated to be 10 μL. A statistically significant zone of inhibition, and the antibacterial zone was more marked in Gs than Gc for *S. mutans* and *S. epidermis* (*p* < 0.001). Tetracycline had less antibacterial activity than lemongrass. Therefore, the herbal EO may be an adjunctive treatment for periodontitis or an alternative to tetracycline.	[303]/2019
	In vitro	*A. naeslundii*, *P. gingivalis*	Gs: *Cymbopogon citratus* EO [MIC was measured]	Based on the results, EO had MIC values of 0.44 and 0.22 mg/mL against *A. naeslundii* and *P. gingivalis*. Both reference strains and most clinical isolates, especially the tetracycline-resistant strains, are sensitive to *Cymbopogon citratus* EO.	[239]/2009
*Eucalyptus globulus*	In vitro	*P. gingivalis*, *F. nucleatum*, *A. a*	Gs: *Eucalyptus globulus* EOs [Their antioxidant capacity and MIC were measured]	In the analyzed oils, the antioxidant activity was weak, although the antibacterial activity was significant, especially against *F. nucleatum* (MIC = 1.14 mg/mL) and *P. gingivalis* (MIC = 0.28 mg/mL). A potential therapeutic application for *E. globulus* EOs may be periodontal disease treatment.	[304]/2015
*Garcinia mangostana*	In vivo	Wistar rats with administered *A. a*	G1: Tetracycline gel (0.7%) G2: Mucoadhesive patch G2: An extract of mangosteen peel applied to a mucoadhesive patch [Histopathological examinations were performed to quantify osteoblasts and osteoclasts]	Osteoclasts and osteoblasts were significantly reduced in all groups by G3 (*p* < 0.05). Mangosteen peel extract inhibited osteoclasts and stimulated osteoblasts, thus preventing alveolar bone damage in periodontitis.	[305]/2021
*Glycyrrhiza uralensis*	In vitro	*P. gingivalis*	Gs: *Glycyrrhiza uralensis* root extract [MIC and MBC were evaluated]	It was found that the licorice root extract had antimicrobial activity against *P. gingivalis* at an MIC value of 62.5 μg/mL and an MBC value of 25 μg/mL. Biofilms of *P. gingivalis* were also affected by licorice root extract. A potential therapeutic application of licorice root extract could be for periodontal disease.	[306]/2017
	In vivo	GCF samples from patients with mild–moderate periodontitis	G1: Doxycycline G2: Licorice G3: Placebo [MMP-8 concentration was measured]	There was a statistically significant difference between G1 and G2 and G3 in the mean MMP-8 concentrations (*p* < 0.001). A statistically significant difference was not detected between G2 and G1 in the mean MMP-8 concentration. Licorice extract is a powerful natural remedy for periodontitis and inflammation, as well as preventing MMPs from being released by the host cells. There were no side effects associated with the use of licorice extract.	[261]/2013
*Glycyrrhiza glabra*	In vitro	Pathogens responsible for plaque colonization and periodontitis	Gs: *Glycyrrhiza uralensis* bark extract [Zone of inhibition was measured]	A potential antibacterial effect was observed for *G. glabra* against primary plaque colonizers and periodontal pathogens (ZOI = 9.2 ± 1.09 and 10.6 ± 0.54 mm, respectively). Statistically, there was no significant difference between *G. glabra* and standard antibiotics for periodontal pathogens.	[307]/2016
*Juglans regia*	In vitro	*G. adiacens*, *S. sciuri*, *Kocuria* spp.	Gc: Ciprofloxacin (5 μg/mL) + cefotaxime (30 μg/mL) Gs: Crude aqueous extracts from *Juglans regia* (100 mg/mL, 250 mg/mL, 500 mg/mL). [Measurement of zone of inhibition]	Compared to the other extracts, the 250 mg/mL extract was more effective. The extract showed the greatest impact on *Kocuria* spp. The extract’s active components increased biological activities, thus aiding in fighting bacterial infections.	[308]/2021
	In vitro	*P. gingivalis*	G1: Immature fruit ethanol extraction G2: Immature fruit methanol extraction G3: Woody parts ethanol extraction G4: Woody stems ethanol extraction G5: Woody stems methanol extraction [MIC, SI, and IC_50_ were measured]	The MIC and SI of the five extracts of *J. regia* studied were as follows: G5 (MIC 64 μg/mL, SI > 16), SI > 16), G4 (MIC 64 μg/mL, SI > 16), G3 (MIC 32 μg/mL, SI > 32), G2 (MIC 32 μg/mL, SI > 32), and G1 (MIC 64 μg/mL.	[155]/2020
	In vitro	*S. mutans*, *S. salivarius*, *S. sanguis*, *S. aureus*	Gc(+): Erythromycin 15 μg + tetracycline 30 μg Gc(-): Water Gs: Aqueous and ethanolic extracts of *Juglans regia* bark [MIC was measured]	Aqueous and ethanolic extracts were found to be the most potent against *S. sanguis* and *S. mutans*, respectively. All strains of bacteria tested were significantly inhibited by the ethanolic extract. An antibacterial effect was not observed on *S. mutans* in the aqueous extract in comparison with the ethanolic extract. In comparison with the control, the aqueous extract significantly inhibited *S. sanguis*, *S. salivarius*, and *S. aureus* (*p* < 0.0001), In comparison with erythromycin, it did not affect *S. mutans*. The growth of oral bacteria was significantly inhibited by ethanolic and aqueous bark extracts of *Juglans regia*.	[290]/2013
*Lippia sidoides*	In vivo	Wistar rats with ligature-induced periodontitis	Gc(+): Diethylammonium diclofenac gel at 10 mg/g Gc(-): Saline gel Gs: Thymol gel [Histopathological analyses were performed]	Compared with a control of saline gel, Gs reduced histopathological lesions in gingival tissue and reduced myeloperoxidase activity (*p* < 0.05).	[309]/2016
Manuka honey	In vitro In vivo	*E. nodatum*, *S. mutans*, *C. rectus*, *S. sangiunis*, *A. a*, *P. gingivalis*	In vitro section (G1: 0.2% CHX, G2: honey mouthwash, G3: saline) [MIC was measured] In vivo section: Plaque regrowth was simulated for four days. Four days after baseline, PI was measured	Among the six microorganisms tested, honey mouth rinses inhibited their growth effectively. All test species showed the lowest MICs with CHX rinses over honey and saline rinses. As a result of in vivo testing, CHX and honey rinses inhibited or reduced plaque formation. Testing showed honey to be antibacterial and antiplaque.	[310]/2012
*Myristica fragrans*	In vitro	Ten tissue samples from patients with chronic periodontitis undergoing a flap surgery	Gc: Doxycycline Gs: Myristica fragrans [Zone of inhibition and antiprotease activity were measured]	*Myristica fragrans*, when added to the tissue sample, showed no zone of clearance compared to a significant zone of clearance of the tissue sample alone. Doxycycline demonstrated a small zone of clearance. *Myristica fragrans* possesses a better antiprotease activity as compared to doxycycline in vitro.	[311]/2016
*Myristica fragrans*	In vitro	*P. gingivalis*	Gs: *Myristica fragrans* extract [Zone of inhibition was measured]	A 13.5 mm inhibitory zone was found in nutmeg extract. *Myristica fragrans* inhibited *Porphyromonas gingivalis*	[312]/2016
	In vitro	*P. gingivalis*	Gs: Isolated malabaricone C from nutmeg (*Myristica fragrans*) [MIC was measured]	Arg-gingipain was irreversibly inhibited by malabaricone C at 0.7 μg/mL, and *P. gingivalis* was selectively inhibited.	[313]/2014
*Ocimum sanctum*	In vitro	*A. a*, *P. intermedia*, *P. gingivalis*	Gc(+): Doxycycline Gc(-): Dimethyl formamide Gs: Ethanolic extract of Tulsi leaves (0.5%, 1%, 2%, 5%, and 10%) [Zone of inhibition was measured]	It was found that Tulsi extracts showed similar inhibition zones to doxycycline at concentrations of 5% and 10%, with similar antimicrobial activity against *A. a* (*p* > 0.05). However, *P. gingivalis and P. intermedia* resisted Tulsi extract, showing significantly smaller inhibition zones (*p* < 0.05). Due to its antimicrobial properties, Tulsi may be used as a complementary therapy to standard periodontal care.	[314]/2016
	In vivo	Wistar albino rats with ligature-induces periodontitis	G1: Control G2: Plain gel G3: 2% *O. sanctum* gel. [GI, PD, and morphometric analysis were performed]	Inhibition of edema by 2% Tulsi (*O. sanctum*) gel was 33.66% at 24 h. The GI and PD demonstrated statistical significance. No significant differences were found between the groups based on the morphometric analysis. Tulsi extract 2000 mg/kg was not found to have any toxic effects when administered orally. The *O. sanctum* gel was effective in treating experimental periodontitis.	[315]/2015
*Pistacia atlantica Kurdica*	In vitro In vivo	*P. gingivalis* Wistar rats	Gs: EO extracted from the gum of *Pistacia atlantica Kurdica* [MIC and MBC were measured; histological analyses were performed]	The experimental gel produced adequate wound healing and exhibited inhibitory and bactericidal activity against *P. gingivalis*.	[316]/2019
*Salvadora persica*	In vitro	*P. gingivalis* and HSV-1	Gs: Miswak raw extract [MIC, IC_50_, and MTT antiviral assays were measured]	An MIC of 62.5 μg/mL was determined against *P. gingivalis*. A therapeutic index of 11.3 μg/mL was observed against HSV-1. A concentration of 18.6 μg/mL was calculated as the IC_50_. A concentration of 210 μg/mL caused cytotoxicity in 50% of Vero cells. The SP films significantly inhibit *P gingivalis* and the HSV-1.	[317]/2020
	In vitro	*S. mutans*, *S. mitis*, *Candida albicans*, *L. acidophilus*, *P. intermedia*, and *Peptostreptococcus*	Gc(+): CHX Gc(-): Distilled water Gs: Aqueous and alcoholic extracts of SP (200 μg/mL and 400 μg/mL) [MIC was measured]	No significant results were obtained when *Salvadora persica*’s water extracts were tested, except for the minimum inhibitory effect against bacteria. *Salvadora persica* alcoholic extract exhibited relatively significant inhibitory effects. On all tested pathogens, alcoholic extract from SP showed antimicrobial activity.	[318]/2016
*Satureja hortensis*	In vitro	*A. a*, *P. gingivalis*, *P. micra*, *T. forsythia*, *F. nucleatum*, *P.* *Intermedia*, *P. nigrescens*	Gc: CHX Gs: *Satureja hortensis* EO [MIC and antibiofilm effects were measured]	All tested bacteria were inhibited by *S. hortensis* EO, despite its low MIC value. All strains of bacteria tested showed inhibition of proliferation at 0.125 µL/mL. In tests against periodontal bacteria, *S. hortensis* EO had limited antibiofilm activity (0.01 µL/mL), inhibiting only *P. nigrescens* biofilm formation.	[319]/2009
*Syzygium aromaticum*	In vitro	*A. a*, *F. nucleatum*, and *P. intermedia*	G1: Hydro-ethanolic extracts G2: Delipidated hydro-ethanolic extracts G3: Fresh extract [MIC, MBC, and zone of inhibition were measured]	According to the MIC values, the tested organisms were antibacterial when tested at 6.25–25 mg/mL. On all bacteria subjected to the extract, the non-delipidated dry extract had a bactericidal effect. *F. nucleatum* was also shown to be bactericidal by delipidated extracts, as well as *A. actinomycetemcomitans* by fresh extracts.	[320]/2021
	In vitro	*P. gingivalis*	Gc(+): Tinidazole Gs: Syzygium aromaticum leaf essential oil (CLEO)-derived eugenol [MIC, MBC, CFU count, SEM, PI uptake, nucleic acid and protein leakage, biofilm quantification, and PCR were performed]	The amount of eugenol in CLEO, 90.84%, was found to have antibacterial activity against *P. gingivalis* at a concentration of 31.25 μM. The presence of eugenol at different concentrations inhibited the formation of biofilms and reduced the preformed ones of *P. gingivalis*.	[148]/2017
*Terminalia chebula*	In vitro	*S. mutans*, *A. a*	Gc: Dimethyl sulfoxide (0.01%) Gs: Ethanol extract of *Terminalia chebula* (EETC) [MIC, susceptibility test, cytotoxicity assay, PGE2 assay, PCR, inflammation antibody array, protease array, ECM degradation, osteoclast formation, and pit formation were studied]	By inhibiting the growth of bacteria, EETC also inhibited the stimulation of PGE2, COX-2, and inflammatory cytokines. In the osteoblasts, EETC stimulated lipopolysaccharide derived from dental plaque to inhibit bone resorption and inhibit osteoclast formation.	[321]/2017
*Vaccinium macrocarpon*	In vitro	*P. gingivalis*	Gc: Phosphate-buffered saline Gs: Cranberry juice concentrate prepared as a non-dialysable material [Growth, adherence properties, and biofilm formation of *P. gingivalis* were studied]	With cranberry concentrations exceeding 62.5 mg/mL, significant inhibition was observed (*p* < 0.05). With cranberry, *P. gingivalis* could not adhere effectively to collagen-, fibrinogen- or human serum-coated surfaces. Cranberry constituents may help prevent and treat periodontitis by preventing *P. gingivalis* from colonizing periodontal sites.	[322]/2006
*Vicia faba*	In vitro	*P. gingivalis*	Gs: *Vicia faba* ethanolic and methanolic extracts [MIC, SI, and IC_50_ were measured]	*Vicia faba* exhibited low cytotoxicity and antibacterial activity.	[155]/2020
*Vitis vinifera*	In vivo	Rats with ligature-induced periodontitis	G1: Laboratory diet G2: GSE for eight weeks G3: GSE for six weeks G4: GSE for two weeks [Histopathological studies were performed to determine ICN, CAL, OD, IL-10, and TGF-β]	GSE groups had lower ICN, higher CAL, and lower OD (*p* < 0.05). In the GSEs and GEs, IL-10 levels were higher (*p* < 0.05). In group B, periodontal ligament IL-10 levels were highest (*p* < 0.05). All groups had higher levels of TGF-ß in the gingival epithelium (*p* < 0.017).	[323]/2017
*Zingiber officinale*	In vitro	*P. gingivalis*, *P. endodontalis*, *P. intermedia*	Gs: Ethanol and *n*-hexane extracts of ginger [MIC and MBC were measured]	The two alkylated gingerols, [10]-gingerol and [12]-gingerol, inhibited oral pathogen growth at MICs of 6–30 μg/mL. At an MBC range of 4–20 μg/mL, these ginger compounds also killed oral pathogens, but not 5-acetoxy-[6]-gingerol, galanolactone, or 3,5-diacetoxy-[6]-gingerdiol.	[324]/2008

**CBC**: Complete Blood Count; **ESR**: Erythrocyte Sedimentation Rate; **ALT**: Alanine Transaminase; **AST**: Aspartate Transaminase; **Gs**: Study Group; **Gc**: Control Group; **MBC**: Minimum Bactericidal Concentration; **EPS**: Extracellular Polysaccharides; **MFC**: Minimum Fungicidal Concentration; **MIC**: Minimum Inhibitory Concentrations; **CBEO**: *Cinnamomum zeylanicum* Bark Essential Oil; **PI:** Propidium Iodide; **SI**: Selectivity Index; **IC_50_:** Half MIC; **MAPK:** Mitogen-Activated Protein Kinase; **EO**: Essential Oil; **MEC**: Ethanol Extracts of *Garcinia mangostana* Peel and Propolis; **ZOI**: Zone of Inhibition; **SEM:** Scanning Electron Microscope; **GCF**: Gingival Crevicular Fluid; **CFU**: Colony-Forming Units; **PI:** Plaque Index; **MSE:** *M. alba* Stem Extract; **LPS**: Lipopolysaccharide; **hPDL:** Human Periodontal Ligament; **GI:** Gingival Index; **PD:** Pocket Depth; **PYC:** Pycnogenol^®^; **SP:** *Salvadora persica*; **HSV**: Herpes Simplex Virus; **MTT**: (3-[4,5-dimethylthia-zol-2-yl)-2,5-diphenyltetrazolium bromide); **CLEO**: *Syzygium aromaticum* Leaf Essential Oil; **EETC:** Ethanol Extract of *Terminalia chebula*; **PGE2:** Prostaglandin E2; **AC-PACs**: A-Type Cranberry Proanthocyanidins; **GSE**: Grape Seed Extract; **ICN:** Inflammatory Cell Number; **CAL:** Connective Tissue Attachment Level; **OD:** Osteoclast Density.

### 4.22. Lippia sidoides

An aromatic *Verbenaceae* shrub known as *Lippia sidoides* is commonly found in the northeastern part of Brazil (where it thrives in semiarid conditions) and is referred to as “Pepper-Rosmarin” [325,326]. The EOs and other types of extracts derived from various parts of this plant contain monoterpenes, such as thymol and carvacrol, which have antimicrobial properties [181,327]. Traditional Brazilian medicine uses *L. sidoides* extracts as a topical antiseptic to treat skin and mucous membrane lesions [328,329]. The use of this plant in dentistry has reportedly produced satisfactory results, particularly in managing supragingival biofilm, as well as antiplaque and antigingivitis effects in humans [180,181,330,331] and animal investigations [332,333,334]. Researchers found that periodontal inflammation could be controlled by reducing pro-inflammatory cytokines such as IL-1β and TNF-α and suppressing gingival neutrophil infiltration by reducing myeloperoxidase activity [332].

### 4.23. Mangifera indica (Mango)

*Mangifera indica* (mango), composed of 10% mangiferin, belongs to the *Anacardiaceae* family. Various medicinal purposes have been attributed to this tropical and subtropical herb [335]. One of the mechanisms involved with mangiferin is that it is a glycosylated xanthone in nature with immunomodulatory and anti-inflammatory properties [336,337]. An experiment showed bone anti-resorption effects in lumbar vertebrae [338]. This plant is also effective against certain periodontal bacteria [339].

### 4.24. Manuka Honey

Since ancient times, honey has treated infections and other medical conditions [340]. Researchers revived interest in honey because of its antibacterial properties, particularly against antibiotic-resistant microorganisms and wound infections [341,342]. This led to manuka honey being approved to treat bacterial infections, ulcers, and burns [343,344,345]. Various proteinaceous substances, the hyperosmolarity effect, an acidic pH, bee defensin-1, hydrogen peroxide, flavonoids, and methylglyoxal, phenolic compounds [343,346,347] are all antibacterial components of honey. However, hydrogen peroxide has the most antimicrobial impact among most kinds of honey [348]. Manuka honey exhibits antibacterial activity, including biofilm and planktonic bacteria [349,350,351]. When cultured as planktonic bacteria [352,353], *P. gingivalis* [354] and *A. a* [355] are sensitive to manuka honey, whereas when developed as a biofilm, *P. gingivalis* is substantially more resilient [356]. English et al. discovered that chewing manuka honey strips reduced plaque development and gingival bleeding [184].

**Figure 9:**
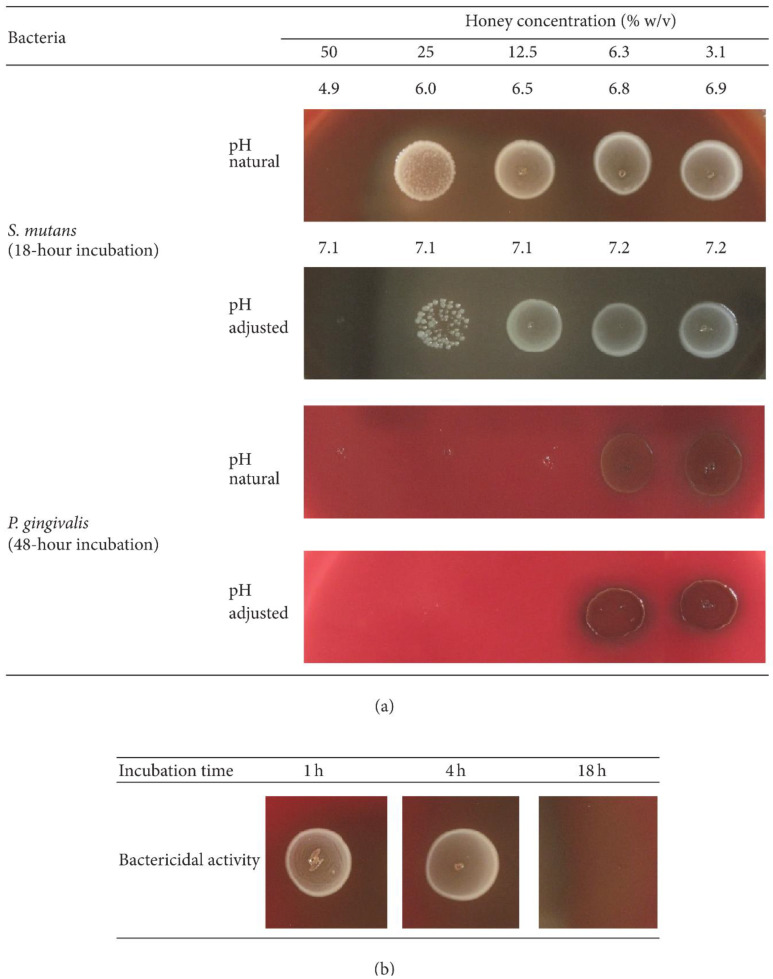
An evaluation of manuka honey’s bactericidal effect (**a**) and effective manuka honey incubation period (**b**) [357].

Safii et al. assessed manuka honey’s antibacterial activity against plaque-associated bacteria to evaluate its potential use in periodontal therapy. The differences between white clover honey and manuka honey were studied at neutral and natural pH levels using the MIC and MBC. Their MBCs were 12.5–50% (*w*/*v*) in manuka honey and between 6.3% to 50% (*w*/*v*) in clover honey. It took 18 h for both types of honey to be bactericidal. The pH-adjusted manuka honey retained its bactericidal activity (Figure 9), while the pH-adjusted clover honey exhibited variable bactericidal effects [357].

### 4.25. Matricaria aurea and Matricaria chamomilla

The ancient medicinal plant *Matricaria chamomilla* (MTC) (chamomile) is an aromatic daisy from the *Asteraceae* family whose flower extracts and oil can be used to treat a variety of ailments [358]. It is rich in active constituents such as spiroether, flavonoids, coumarins, and terpenoids [358,359,360]. Spigenin, chamazulene, and bisabolol are the anti-inflammatory components of MTC extract, inhibiting NO generation, hyaluronidase, collagenase, cyclooxygenase prostaglandin E2, interleukins (1β, 6, 12), and TNF-α [361,362]. It has been shown that using MTC oral rinse improved plaque buildup, gingival irritation, and recurrent stomatitis [186,363,364,365,366]. *Matricaria aurea* (*M. aurea*), a plant native to Saudi Arabia that belongs to the genus *Matricaria*, has recently been studied for its therapeutic effects and potential to be a rich source of antimicrobials and antioxidants [367,368]. *Matricaria chamomilla* shares many similar chemical characteristics with this species [369]. Ahmad et al. revealed recently that this extract could be a source of numerous substances that may benefit the development of the next phase of the drugs used in treating chronic periodontitis [370].

### 4.26. Morus alba (M. alba)

*Morus alba* has traditionally been used to cure fevers, enhance eyesight, strengthen joints, and decrease blood pressure [371]. Mulberry fruit is also used to cure weakness, exhaustion, anemia, and premature hair greying, to replenish the blood, and to aid the kidneys. Mulberry root bark possesses anti-inflammatory, hypoglycemia, and antibacterial, antibacterial qualities, and it exhibits anti-inflammatory capabilities [372]. The ethanolic extract of the stems of the mulberry tree contains the most oxyresveratrol (2,3′,4,5′-tetrahydroxystilbene), whereas the ethanolic extract from the leaves contains the least [373]. *M. alba*’s oxyresveratrol has antioxidant and radical-scavenging properties. Several components, including prenylated flavonoids, may have anti-inflammatory effects by preventing the generation of nitric oxide (NO) [374]. In several studies, *M. alba* has been reported to be beneficial for treating periodontitis (Table 1 and Table 2).

### 4.27. Myristica fragrans (Nutmeg)

*Myristica fragrans* belongs to the family *Myristicaceae* and is frequently planted for spices on Malaysia’s Penang Island. Its four components are skin, flesh, seeds, and mace [375]. *Myristica fragrans* contains a variety of alkyl benzene derivatives [376,377]. Scientists from many fields have researched the chemical constituents of *Myristica fragrans* for their hypolipidemic and hypocholesterolemic, antimicrobial, antidepressive, and antioxidant characteristics, etc. [375]. The seed kernel possesses antithrombotic, antiplatelet, and antifungal properties, among others [378], while the mace has antipapillomagenic, anticarcinogenic [379], and anti-inflammatory activities [380].

Trimyristin, a chemical derived from the seeds of *Myristica fragrans*, has been shown to have antibacterial effects against Gram-negative and -positive bacteria [381]. In extracts of the flesh, seeds, and mace of *Myristica fragrans*, Zaleha Shafiei et al. found decreased bacterial concentrations [382]. Nutmeg’s essential oil contains the 5-lipoxygenase inhibitors limonene, β-pinene, α-pinene, and sabinene [383]. Limonene is a specific COX-2 inhibitor with notable inhibitory effects on PGE2 synthesis [384]. Terpene-4-ol, found in seed oils at 7.2% and mace oils at 23.6%, inhibits the production of IL-10, IL-1β, TNF-α, IL-8, and PGE2 [385]. Although it does not decrease IL-1β, α-pinene lowered the pro-inflammatory IL-6 produced in the mouse colon [386]. Sabinene, eugenol, and α-pinene prevent the synthesis of TNF-α [387]. Additionally, sabinene blocks IL-1β and IL-6 [383]. The presence of macelignan also inhibits the expression of MMP-9 [388]. *Myristica fragrans* may be a supplemental treatment for periodontitis due to its anti-inflammatory characteristics [389]. It is still necessary for further research to determine whether *Myristica fragrans* can act as an anticollagenolytic agent in periodontitis.

### 4.28. Ocimum sanctum (Tulsi)

**Figure 10:**
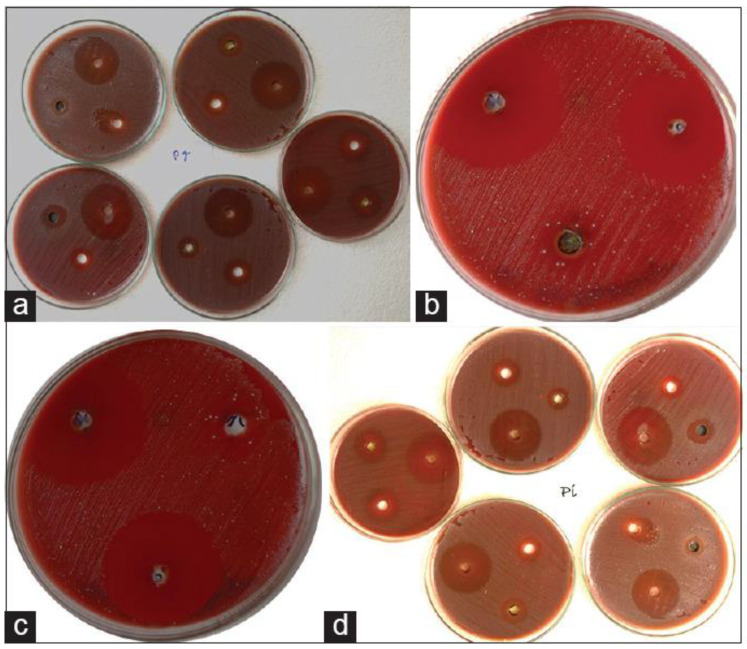
Different concentrations of Tulsi on growth inhibition of *P. gingivalis* (**a**); 5% Tulsi against *A.a* (**b**); 10% Tulsi against *A. a* (**c**); and against *P. intermedia* (**d**) [314].

Holy basil, or Tulsi or *Ocimum sanctum*, is an aromatic plant from the *Labiatae* family. This tiny plant is used in terms of its various parts for its medicinal properties. It has antioxidant abilities due to its COX2 inhibitory functions and the ability to protect against radiation poisoning and cataract formation [390,391,392]. In periodontal diseases, it has been demonstrated to have antimicrobial [393], antioxidant (by scavenging free radicals through phenolic compounds such as cirsilineol, apigenin, rosmarinic acid, and eugenol), and antigingivitis (due to the presence of compounds such as civsilineol, civsimavatine, isothymonin, apigenin, rosavinic acid, and eugenol) properties by inhibiting COX activity [394]. Tulsi’s methanolic and aqueous extracts are also pronounced analgesics, antipyretics, and anti-inflammatory agents [395,396]. The findings of a study on the antibacterial effects of Tulsi extract and doxycycline on periodontal microorganisms are shown in Figure 10. Tulsi extracts at 5% and 10% had doxycycline-like antibacterial action against *A. a*; however, *P. gingivalis* and *P. intermedia* had markedly greater resistance to Tulsi extract [314].

### 4.29. Pinus pinaster

Pycnogenol^®^ (PYC), a standardized bark extract, is produced from the French maritime pine *Pinus pinaster* (previously *Pinus maritime*), which is found along the southwest French coast. Procyanidins constitute 65–75% of PYC extract. Procyanidins themselves are composed of catechins and epicatechins. Other compounds include phenolics, cinnamic acids, glycosides, and polyphenolic monomers [397]. Studies have shown that the extract and specific fractions have potential antioxidant properties in cultured cells, perfused organs, and when used in vivo [398,399]. The anti-inflammatory activities of the PYC compound have been demonstrated in human studies [397,400,401]. One study showed chewing gum with PYC may prevent gingival bleeding and plaque formation [402].

### 4.30. Piper marginatum and Ilex guayusa

*Piper marginatum* may be found in the Caribbean from Guatemala to Brazil. It belongs to the *Piperaceae* family. There are also names for this plant in Colombia, including “small cord” and “tooth healer” [403,404]. Against diseases in humans, animals, and plants, *P. marginatum* leaf extracts have shown antibacterial, antimycotic, and antiviral properties [403,404]. Guayusa is the common name for “*Ilex guayusa*”, an *Aquifoliaceae* plant that grows in tropical and subtropical climates [405]. When its leaves are consumed in infusions, they stimulate nerves and muscles and treat colds, respiratory problems, and gastrointestinal disorders [405]. Gamboa et al. reported the antibacterial properties of the extract of these plant leaves against microorganisms involved in periodontal diseases [406].

### 4.31. Pistacia lentiscus (Mastic Gum)

**Figure 11:**
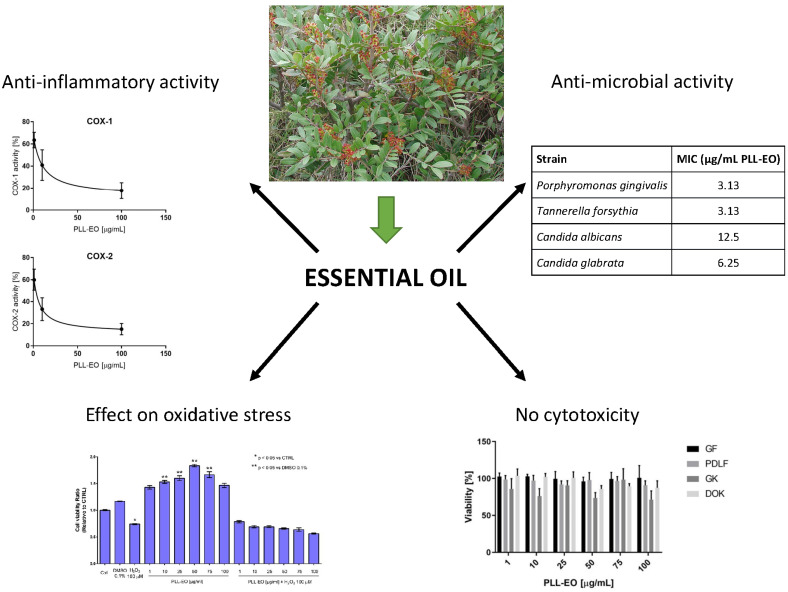
Anti-inflammatory, oxidative stress, cytotoxicity, and antimicrobial characteristics of using *Pistacia lentiscus* essential oils against periodontal bacteria and *Candida albicans* (GK: gingival keratinocytes, PDLF: periodontal ligament fibroblasts; GF: gingival fibroblasts; and DOK: dysplastic oral keratinocytes) [414].

*Pistacia lentiscus* (PL), a member of the *Anacardiaceae* family and a species native to the Mediterranean region that is primarily found in Sardinia (Italy), is a wild-growing plant [407]. *Pistacia lentiscus* plant and processed products were traditionally used for their antiseptic, anti-inflammatory, and analgesic properties [407,408]. *Pistacia lentiscus* has antibacterial and antifungal properties throughout many applications [409]. Several volatile chemicals in mastic gum include α-pinene, β-caryophyllene, β-myrcene, and limonene [410]. Triterpenic acids are the principal active components of mastic gum, which has strong bactericidal properties, especially against *H. pylori* [411]. A recent study demonstrated that mastic gum is effective against anaerobic oral infections such as *F. nucleatum*, *P. intermedia*, *and P. gingivalis* [278]. Likewise, the resin of *Pistacia lentiscus* is used to make dental powder, which is used to eliminate bad breath and to clean teeth [14]. However, due to its limited solubility, it may be more effective for local application as opposed to mouthwash [412]. Additionally, *Pistacia vera* extracts have inhibited adhesion and glycolysis in oral *streptococci* [413]. An EO of PL leaves showed antimicrobial activity against periodontal bacteria. COX-1/2 and lipoxygenase were tested to investigate the anti-inflammatory activity, while the antioxidant capacity was assessed electrochemically and by an MTT assay. As a result of COX-1/2 inhibition, PL EO was shown to have anti-inflammatory activity in a dose-dependent manner (Figure 11) [414].

**Figure 12:**
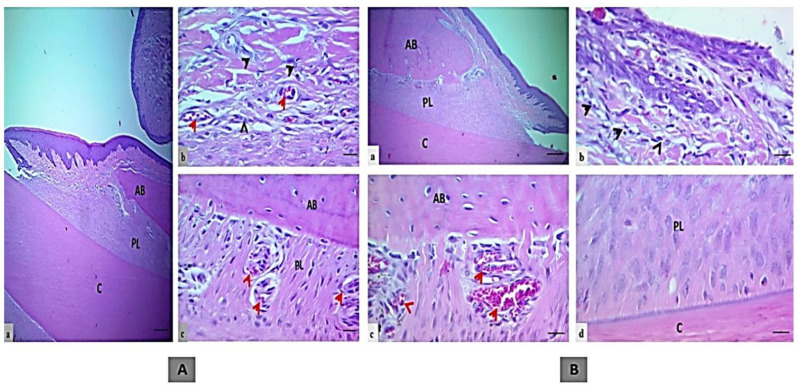
A histological section of a rat incisor tooth and its surrounding periodontal tissues. Treatment control group (**A**): mild inflammatory cells (black arrows) with intact junctional epithelium and a stable bony surface with dense, well-formed bone and multiple blood vessels (red arrows) (H&E, scale bar 10 μm in section (**a**), and 20 μm in section (**b**,**c**)). Testing group (**B**): mild inflammatory cells (black arrowheads) with intact junctional epithelium and well-formed, dense bone (H&E, scale bar 10 μm in section (**a**), and 20 μm in section (**b**–**d**)) (AB; alveolar bone, PL; periodontal ligament and C; cementum) [415].

Experimentally induced periodontitis in rats was treated with EO of *Pistacia atlantica kurdica* gel and its effect on osteoclastogenic bone markers examined in a study by Azeez et al. H&E slides were processed from the mandibular central incisors 30 days after sacrifice, and histologically, the inflammation, osteoclasts, and PDL were examined. An ELISA was also used to measure the RANKL and IL-1β concentrations. Despite the presence of mild inflammation, the junctional epithelium was intact. In addition, the bone form had a regular shape and good density. As shown in Figure 12A, the PDL space was wide and filled with proliferating ligament tissue attached to a normal cementum layer. The control group filled the PDL space with less organized proliferating PDL tissues, as demonstrated in Figure 12B [415].

### 4.32. Psidium guajava (Guava)

This tiny tree may reach 20 feet in the Amazon rainforest [416]. The opposite, oblong leaves range in size from three to seven inches, and the undersides have noticeable veins. It has white flowers that are about an inch wide. Pear-shaped, oval, or spherical fruit are produced by *P. guajava* (guava). Two varieties exist, one with a thin shell and numerous seeds encapsulated in a solid pulp and one with a thick shell and few seeds [417]. Guava is an antiplaque, anti-inflammatory, antioxidant, and wound-healer agent. A paste made from fragile guava leaves is traditionally used to improve oral hygiene [418].

In particular, the flavonoids guaijaverin and quercetin have been linked to the antibacterial action of guava against bacteria [419,420,421]. The bark also has antimicrobial qualities because of tannins [422]. There has been evidence of the inhibitory effects of quercetin on *P. gingivalis*, *P. intermedia*, *F. nucleatum*, and *Actinomyces* species. Quercetin acts against bacteria by forming irreversible complexes that disrupt membranes and inactivate extracellular proteins [423,424]. As a result of its antiplaque properties, guaijaverin derived from guava leaves inhibits *S. mutans* and *S. aureus* [418,425,426,427]. Guaijaverin reduces the ability of pathogenic oral bacteria to attach to the tooth surface due to their reduced hydrophobicity [418] by binding to cell surface proteins and decreasing the total hydrophobicity of a cell, suggesting that guava might be developed as a natural antiplaque agent [418]. Researchers have shown that mouthwash containing an aqueous extract of the leaves is particularly efficient in preventing the proliferation of *S. aureus* and *E. coli* bacteria [428]. Gingivitis was reduced significantly after using guava leaf extract in a mouthwash [429]. Guava’s cytotoxic ability would make it more advantageous to use it as an additive in manufacturing products for oral health care [430]. Therefore, guava might complement conventional periodontal treatment with its antibacterial and antiplaque properties.

Guava’s anti-inflammatory properties come from its capacity to suppress prostaglandin, kinin, and histamine [431]. It was found that guava extract completely neutralized the cytotoxic, pro-inflammatory reaction caused by *A. a* leukotoxin [432]. In addition to their anti-inflammatory properties, guava leaf and stem extract lower CRP levels, modulating the inflammatory response [433]. Additionally, guava’s immunomodulatory activity on NF-kβ has been demonstrated [434]. Periodontal disease can be treated with guava by blocking NF-kβ, inducible NO synthase, and COX-2, suggesting an effective way to reduce inflammation-induced bone resorption [435,436].

Periodontal inflammation may also trigger excessive production of free radicals from neutrophils, resulting in tissue destruction [437]. Diets rich in antioxidants have been suggested to prevent and manage periodontal diseases [438,439]. Antioxidant micronutrients are vital to reduce excessive cytokine production and control oxidative and tissue damage [440]. In addition to being rich in vitamin C, guavas are also excellent antioxidants [441,442]. The quercetin, carotenoids, vitamin C, and polyphenols in guava are responsible for the antioxidant effect [438,443,444]. Consequently, guava might be used to treat periodontal diseases using antioxidant-based mechanisms.

Fibroblasts characterize periodontal connective tissue as the most common cell type. Collagen fibers compose the periodontal and gingival ligaments [445]. The effect of ascorbic acid on the extracellular matrix modulates procollagen gene expression, which leads to collagen synthesis [446]. Guava extracts may facilitate tissue healing [447], as they are a potential source of vitamin C and bioflavonoids [448]. In addition to maintaining the functional and structural integrity of the epithelium and the physiologic or metabolic parameters important for periodontal health, vitamin C also supports immunological function [449]. Swollen gums are recommended to be treated with a mouthwash produced from its root bark, while swollen and bleeding gums could be treated with a gargle made with a decoction of leaves [450].

### 4.33. Punica granatum (Pomegranate)

*Punica granatum* is the scientific name for pomegranate, which belongs to the *Punicaceae* family [451]. It is characterized by its spiny branches, lance-shaped glossy leaves, gray aged barks, large red or white flowers, ripe fruit that has a leathery and deep red skin five inches wide and with a grenade shape with a pointy calyx, and seeds that are enclosed in a white, membranous pericarp. A tart, red liquid surrounds each seed [452]. Due to their benefits, pomegranates are considered “a pharmacy unto themselves” [452]. Pomegranate offers a variety of possible health benefits, including those for bacterial, fungal, viral, immunological, vermifuge, stimulant, refrigerant, stomachic, styptic, diuretic, and helminthic infections [452]. Punicic acid, the primary component of pomegranate fatty acids, is an excellent anti-inflammatory agent, inhibiting prostaglandin synthesis [453]. Both cyclooxygenase (COX-1, COX-2) and lipoxygenase enzymes (critical enzymes in producing several inflammatory mediators) have been inhibited in vitro by cold-pressed pomegranate seed oil [452], and they are also inhibited by orally ingesting the extract of the pomegranate fruit rich in polyphenols [454]. Numerous polyphenolic substances, including ferulic acid, ellagic acid, chlorogenic acid, punicalagin, gallic acid, punicalin, epicatechin, caffeic acid, catechin, delphinidin, and rutin, are present in pomegranate fruit [455]. The fruit extract inhibits IL-1β-induced tissue destruction and the expression of matrix metalloproteinase (MMP) [452]. In addition, it reduces NO and PGE2 production [456]. In addition to the methods above, pomegranate’s anti-inflammatory effects may also result from its immunoregulatory effects on macrophages, T lymphocytes, and B lymphocytes [457]. It can prevent inflammation-induced bone resorption as another mechanism in treating periodontitis by blocking the NF-κB signaling pathway, generating many damaging factors [435,458]. Gingival bleeding was significantly reduced when pomegranate-based dentifrices were used [454]. Macrophages may be protected from oxidative stress and lipid peroxidation by the free radical-scavenging properties of pomegranate extract [452]. Pomegranate flavonoids, which have antioxidant potential, are thought to be responsible for the fruit’s antigingivitis effects [459].

Delivering *Punica granatum* extracts locally after SRP demonstrated that the pocket depths [433] and clinical attachments were improved, and the levels of IL-1β and IL-6 were reduced compared to the baseline [460]. In addition to enhancing oral health, pomegranates may lower the risk of gingivitis. When mouthwashes with dissolved pomegranate extract were used three times a day, antioxidant activity was increased, while aspartate aminotransferase activity (an indicator of cell injury, elevated in periodontitis) decreased [461,462]. A correlation exists between the saliva protein levels and plaque-forming bacterial content in periodontitis patients. The protein levels decreased significantly in saliva samples obtained after using pomegranate mouthwash, indicating antibacterial activity [463]. It has been demonstrated in vitro that pomegranate flavonoids have antibacterial properties [464]. In the formation of dental plaque, *S. sanguis* is thought to be the first colonizer [465] and was shown to be sensitive to pomegranate extract, which has a prevention effect comparable to CHX [466]. The antibacterial action of tannins is thought to be the main reason for their ability to enhance bacteriolysis and interfere with bacterial adhesion processes [466]. The extract of the pomegranate’s fruit bark has also shown promising results compared to CHX in inhibiting the plaque-former bacteria [467]. Compared to CHX, mouth rinses containing pomegranate hydroalcoholic extract reduced the plaque-forming bacteria’s colony-forming units by 84% in 60 healthy individuals. The plaque-forming bacteria’s ability to adhere to tooth surfaces decreased, suggesting that the extract can be beneficial in preventing and treating tooth plaque [468].

Gels containing 10% *Punica granatum* extract were shown to be ineffective in reducing dental plaque and gingivitis [451]. However, combined with mechanical debridement, pomegranate gel improved the clinical and microbiological markers in gingivitis [469]. In 92 subjects, the plaque, gingival, and bleeding indices improved significantly after using pomegranate toothpaste [470]. Its antibacterial properties may make pomegranate a great addition to traditional periodontal care as a plaque-fighting agent [471]. Patients with periodontitis have higher levels of *H. pylori* in deep pockets [472,473,474]. The antibacterial properties of pomegranate have also been demonstrated against *Helicobacter* [475]. The herpes virus-fighting properties of pomegranate extract have also been discovered [453]. Recent research suggested that herpes viruses may cause periodontal damage. Herpes viruses can induce and accelerate periodontitis by triggering the release of cytokines from the cells, disrupting host defense systems, and enhancing the virulence of local periodontal bacteria [458]. Thus, the antiviral properties of pomegranate make it a potential treatment for periodontitis. According to a recent study, pomegranate peel extract reduced the growth of *Trichomonas tenax*, and it can be used to treat acute ulcerative gingivitis [476]. Enteric probiotic microorganisms also benefit from pomegranate administration [477]. Several *Bifidobacterium* and *Lactobacillus* species have developed increased growth in their presence [478]. By affecting the development, adhesion, and colonization of the pathogenic bacteria responsible for periodontitis, decreasing the level of interleukins and MMPs, and improving the epithelium barrier’s role in resisting infections and bacterial invasion, these probiotic species have shown therapeutic properties in treating periodontitis [479,480,481,482]. Combined with methicillin-resistant *staphylococcus aureus* (MRSA)-fighting antibiotics, pomegranate extract has synergistic activity [483]. The added effect of pomegranate peel’s methanolic extract combined with ciprofloxacin was improved regarding the antibacterial activity [484]. Due to its ability to improve antibiotic sensitivity, pomegranate may be an effective treatment for periodontitis.

### 4.34. Rosmarinus officinalis

A little aromatic bush from the *Lamiaceae* family, *Rosmarinus officinalis* is also referred to as “Alecrim” (rosemary) in Brazil. It is a native of the Mediterranean region. It has several therapeutic potentials, including antioxidant, antimicrobial, and antifungal properties. Several components of its leaves, including terpenoids, flavonoids, phenols, and essential oils, contribute to the plant’s characteristics [33,55,485,486]. An investigation by Santoyo et al. showed the EOs responsible for the antimicrobial effect. They concluded that borneol produced better results in terms of antibacterial activity, followed by camphor and verbenone [487]. Lee et al. studied rosmarinic acid in bone cells. They found that it significantly induced alkaline phosphatase activity and enhanced mineralization in osteoblasts, suggesting that it could be used to prevent bone destruction [488].

### 4.35. Salvadora persica (Miswak)

This chewing stick is also called the Miracle Twig or Brushtree in the Muslim world, and it is also known as Miswak, Arak, Meswak, or the Toothbrush Tree [192]. The WHO’s recommendation allows the use of fibrous branches of *Salvadora persica* (SP) in oral hygiene because of its antimicrobial properties [489]. *Salvadoraceae* plants are found in this plant family [490]. The plant is mainly found in Africa’s arid and subtropical regions, the Middle East, and the Indian subcontinent [491]. As a predecessor of toothbrushes, SP was used by Arab and Islamic communities throughout the pre-Islamic and Islamic periods to clean the teeth and improve oral hygiene [492]. When used for brushing, SP’s beneficial properties for dental and oral health result from its mechanical action and pharmacologic active ingredients. An example of its chemically active compounds is tannins, which reduce plaque and periodontal disease by blocking the glucosyltransferase enzyme [493].

Additionally, several compounds found in its natural extracts, including vitamin C, potassium and sodium chloride, silica, salvadorine, salvadourea, saponins, and various minerals, have been linked to SP’s antibacterial, anti-inflammatory and antioxidant properties [493,494]. Studies have examined the efficacy and benefits that SP may have for periodontal care and the mechanism of action through its anti-inflammatory [493,495,496], antioxidant [494,495,496,497,498], and antibacterial effects [493,499,500], as well as the regenerative modulatory activity it has. SP’s clinical therapeutic impact on periodontal health and inflammation (Table 1 and Table 2) has been reviewed [192,193,194,195,501,502,503,504,505,506,507,508,509]. All trials that employed SP (Table 1) showed a substantial decrease in gingival inflammation and plaque buildup, suggesting the effectiveness of SP herbal supplementary treatment in treating or preventing inflammation and plaque, which are significant contributors to periodontal diseases [510]. Comparing the clinical outcomes of SP to those of chlorhexidine (CHX) as a principal mouthwash used in periodontal therapy [511] showed that SP provided equal or better results [507,508]. Moreover, Rezaei et al. discovered that herbal mouthwash considerably reduced GI more effectively than chlorhexidine (*p* < 0.05) [508]. However, Prasad et al. [507] reported that neither 0.2% chlorohexidine nor herbal mouthwash significantly affected GI or PI (*p* = 0.969 and 0.427, respectively).

**Figure 13:**
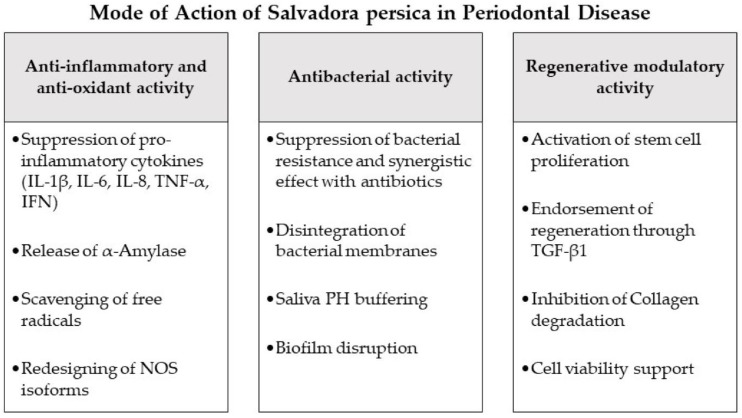
The chemotherapeutic effects of *Salvadora persica* on periodontal diseases [517].

SP extract showed potent antimicrobial and bactericidal effects on the periodontal pathogens examined, especially on Gram-negative bacteria, including *P. gingivalis* [512,513,514]. It was more effective in organic solvent extracts than in water extracts [21,318,515], and it had a coactive mechanism when administered with antibiotics [21] (Figure 13, Table 1 and Table 2). It has also been suggested that SP produces phytochemicals such as β-sitosterol, which may help prevent the formation of genotoxic bacteria compounds on teeth [516]. Moreover, dissolved anionic compounds may damage bacterial cell walls, inhibit oxygen absorption, and cause severe oxidative stress, leading to toxicity [516]. The essential and volatile oils and nonpolar chemicals in SP have also been demonstrated to buffer saliva pH, decrease bacterial activity, and break down plaque and biofilm [516]. According to reports, the antibacterial action was dose- and time-dependent, with the most potent effects occurring right after SP application [512]. *Salvadora persica*’s modes of action in periodontal disease are summarized in Figure 13.

In addition, SP’s diverse effects may affect periodontal therapy and encourage regeneration and repair [496,518,519,520]. According to researchers, SP may increase regenerative and stem cell activities by stimulating the expression of transforming growth factor-1 [496,521]. SP increased mesenchymal stem cells, fibroblast proliferation, and survival, and it reduced collagen degradation, a fundamental cause of periodontal diseases [519,520,522]. Other research found that applying SP extract to defects improved the healing capacity [497,518]. These findings imply that when SP is combined with periodontal therapy, regeneration may significantly speed up. In vivo and in vitro investigations are still required, especially regarding PDL-derived stem cells (Table 2).

### 4.36. Satureja hortensis (Summer Savory)

The annual herbaceous crop species known as summer savory (*Satureja hortensis*) is heavily branching, has linear leaves, and is a member of the *Lamiaceae* family. The principal biomolecules in *S. hortensis* extracts and EOs, including phenolic compounds, pyrocatechols, mucilage, tannins, flavonoids, steroids, volatile oils, acids, and gums, have a variety of antioxidant, antimicrobial, and anti-inflammatory potentials used in treating some systemic diseases [523]. According to Gursoy et al.’s research, *S. hortensis* EO, when applied in a dose safe for keratinocytes, had a marginal antibiofilm effect at subinhibitory levels. Still, it inhibited periodontal bacterial growth [319].

### 4.37. Syzygium aromaticum (Clove)

The dried blossom buds of the clove tree, *Syzygium aromaticum* (*Myrtaceae* family), are used to make the spice clove [524]. They are composed of approximately 14–20% EOs [524]. Many pathogens and bacteria that cause tooth decay and periodontal diseases are sensitive to clove [525]. Additionally, investigations have shown *Syzygium aromaticum* has antifungal, anticarcinogenic, antiallergic, and antimutagenic properties [147,526]. Among the main components of clove oil is eugenol, which is both an antioxidant and anti-inflammatory agent [524,527]. Clove is antibacterial against *P. gingivalis* and *P. intermedia*, two Gram-negative anaerobic infections of the periodontal pockets [525]. The NF-κB signaling pathway may be modulated, and IL-6, COX-2, and TNF-α are suppressed by clove, which may reduce periodontal inflammation [528,529,530,531]. The anti-inflammatory properties of cloves are also accompanied by their antioxidant properties [532,533]. Oxidative stress is joint in periodontal diseases, which could be reduced by its antioxidant potential [532,533,534]. Furthermore, Karmarkar et al. found that dried clove buds are high in eugenol and that its derivatives prevent bone loss, which are favorable properties for treating periodontal diseases [535].

### 4.38. Terminalia chebula

In addition to its anti-inflammatory and antioxidant actions, it has been claimed that the plant *Terminalia chebula* (family *Combretaceae*) has antibacterial and anticariogenic capabilities. The fruit of this plant have been utilized to prevent and treat dental caries, gingivitis, and stomatitis, and mouth rinses containing this agent have been shown to have antibacterial properties against oral pathogens [536]. Numerous phytochemical compounds, such as polyphenols, terpenes, anthocyanins, flavonoids, alkaloids, and glycosides, appear responsible for these positive effects [537]. An essential component of Ayurvedic medicine is the “Triphala” formulation [538]. Triphala’s bulk powder, pill, or liquid extract is used for oral and dental diseases [539].

**Figure 14:**
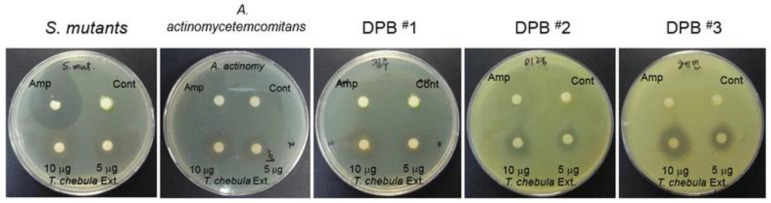
A comparison of the effect of *Terminalia chebula* ethanolic extract and ampicillin (Amp) on the growth of *A. a*, *S. mutans*, and other dental plaque bacteria (DPB#1, DPB#2, and DPB#3) [321].

Additionally, it can heal wounds when applied externally [540]. A key component of Triphala’s treatment for periodontal disease is its anticollagenase activity. Degradation of periodontal tissues occurs in part due to MMPs. Doxycycline inhibits collagenases and gelatinases more effectively than any other tetracycline. On the other hand, tetracycline is not an ideal medicine for long-term use. It is impossible to experience the same adverse effects of tetracycline-like side effects from using herbal extracts to treat periodontal disease. Triphala significantly inhibits PMN-type collagenase, especially when MMP-9 presents in high concentrations (1500 μg/mL) [541]. Triphala is also used as a periodontal mouthwash due to its broad range of activities. It is also suggested to gargle with Triphala for oral diseases. Compared to CHX after oral prophylaxis, Triphala mouthwash after SRP significantly reduced the PI, GI, and oral hygiene index with no sign of tooth discoloration [542]. When taken with 400 mg of metronidazole (TID), Triphala mouthwash ought to be applied BID. Reduced tooth mobility, PD, bleeding, sensitivity to heat and cold, and calculus formation were among the clinical indices that indicated improvements in the results, with minimum recurrence in all of the clinical parameters when taken in this manner, as opposed to those exhibited by 0.2% CHX (BID) + metronidazole 400 mg (TID) [543]. The findings of a study utilizing an ethanolic extract of *Terminalia chebula* and ampicillin to reduce bone resorption and inflammation brought on by dental plaque bacteria are shown in Figure 14. The extract suppressed the growth of *A. a*, dental plaque bacteria, and *S. mutans* [321].

### 4.39. Vaccinium macrocarpon

**Figure 15:**
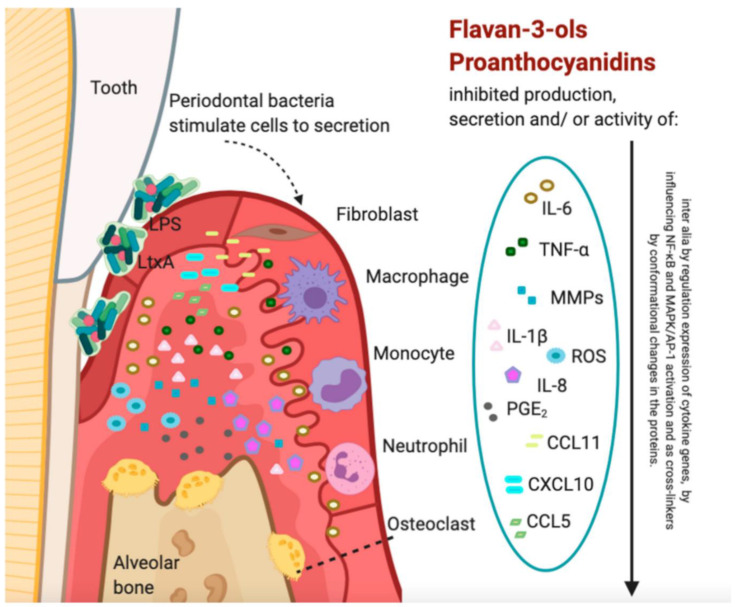
An illustration of flavan-3-ols and proanthocyanidin immunomodulatory activities in periodontitis [550].

The native North American fruit, the cranberry (*Vaccinium macrocarpon*), has become increasingly popular due to its health benefits. The cranberry is a rich source of bioactive flavonoids, such as anthocyanins, flavanols, and proanthocyanidins, making it a potentially useful medicinal herb. It has been demonstrated that cranberries inhibit *P. gingivalis* and *F. nucleatum* colonization in the gingival crevice and prevent *P. gingivalis* from adhering to various proteins, including type I collagen, hence lowering bacterial coaggregation in periodontal disorders [322,544]. Several studies have also demonstrated that cranberries inhibit the red complex’s proteolytic activity [258]. The host macrophages release pro-inflammatory cytokines after being stimulated with lipopolysaccharides derived from bacteria (Figure 15). Bodet et al. argued in 2006 that these cytokines were inhibited [545] since the activator protein 1 (AP-1) regulation was decreased by a fraction when it interfered with the cellular signaling proteins [258,545]. MMPs and elastase, which are involved in the breakdown of tissues, are created by inflammation-producing cells [546]. With the arrest of MMP1 and MMP9 catalytic activity following treatment with the cranberry fraction, the phosphorylation of intracellular kinases was reduced and NF-kβ.P65 activity was suppressed [547]. In regulating fibroblast inflammatory responses in aggressive periodontitis, cranberry components prevent NF-κB and MMP-3 [548]. Tanabe studied the effects of A-type cranberry proanthocyanidins (AC-PACs) on bone resorption and osteoclast development. It was shown that AC-PACs at high concentrations were not toxic to osteoclastic cells. The physiology and maturation of osteoclastic cells can be affected by AC-PACs, which raises the possibility that they might prevent bone resorption [549].

### 4.40. Vicia faba

In the Mediterranean and the Far East, these plants are primarily used as cattle feed rather than human food or as soil nitrogen enhancers [551]. *Vicia faba* is divided into three types based on seed size: var. minor, var. quina, and var. major [552]. Fiber, vitamins, and antioxidants in broad fava beans lower triglycerides and cholesterol [553,554]. Proteins in *Vicia faba* reach their isoelectric point at pH 4.0, where their solubility is the lowest. However, as the pH increases, the solubility rises steadily until it reaches a maximum of 8.0 [555]. The primary components of *Vicia faba* are flavan-3-ols, including catechin and epicatechin, flavonols, and flavones, and all of which contribute to the plant’s anti-inflammatory and antioxidant capabilities [556]. Despite preliminary study findings, several publications provide data about periodontal disorders [557].

### 4.41. Vitis vinifera

Natural antioxidants are abundant in the byproducts of *Vitis vinifera* [202]. Grapes are one of the most significant fruit crops in the world, producing more than 79 million tons of fruit annually [202]. In winery residues, bioactive chemicals have been discovered to possess anti-inflammatory, antioxidant, and cytoprotective properties [558,559]. Most of the health advantages of vine extracts are attributed to phenolic compounds, which are regarded as the most significant active chemicals. Numerous studies have noted that phenolic compounds have antibacterial, antifungal, and antiviral activity, either directly against oral infections or by inhibiting virulence factors [560,561].

Additionally, it has been demonstrated that *V. vinifera* extracts can control the bacterial-induced inflammatory response and oxidative stress imbalance in periodontal diseases [562]. Grape seed extract (GSE) is the most researched byproduct of *V. vinifera*. Furiga et al. discovered that GSE had antibacterial action against two anaerobic bacteria linked to periodontal diseases [563]. It has been demonstrated that human PDL cells exposed to *P. gingivalis* may produce fewer pro-inflammatory cytokines when treated with the phenolic acid resveratrol [564], which was found to decrease IL-1β, IL-6, IL-8, and TNF-α levels as well as alveolar bone loss in an animal model of periodontitis [302]. Resveratrol significantly improves periodontal health, according to research conducted on human volunteers to evaluate the benefits of resveratrol supplementation in diabetic patients [565].

Additionally, Özden et al. revealed GSE as an essential player in periodontal inflammatory processes using histomorphometric and immunohistochemical analyses [323]. Inflammation levels were lower, connective tissue levels were improved, and bone healing was higher [323]. Cranberry proanthocyanidins have also helped treat periodontitis by inhibiting MMPs and pro-inflammatory cytokines, biofilm formation, *P. gingivalis* adhesion to various proteins, and growth of pathogenic bacteria in periodontal pockets [566].

### 4.42. Zanthoxylum armatum

*Zanthoxylum armatum* is a member of the *Rutaceae* family. Its distribution is from Kashmir to Bhutan, at up to 2500 m in altitude [567]. Several species of *Zanthoxylum* are used to improve oral health [568,569]. Several plant parts of *Zanthoxylum* species contain abundant alkaloids, sterols, phenolic acids, lignins, and terpenoids. *Zanthoxylum armatum* bark powder relieves gingival bleeding when mixed with honey. Inflammatory tooth pain is also treated using an extract from *Zanthoxylum*, which is known as the toothache tree [570]. EOs isolated from *Zanthoxylum armatum* leaves were active against all the tested bacterial strains [571]. Due to their terpenoids, *Zanthoxylum armatum* EOs may have antibacterial properties [572].

### 4.43. Zingiber officinale

One medicinal plant used in many nations is ginger (*Zingiber officinale*), which belongs to the *Zingiberaceae* family [573]. Ginger’s active compounds, including β-bisabolene, shogaol, gingerol, and paradol, have glycemic-controlling, anti-inflammatory, antioxidant, anticancer, and antiobesity properties [574,575]. The effectiveness of herbal mouthwash containing hydroalcoholic extracts of *Z. officinale*, *Rosmarinus officinalis*, and *Calendula officinalis* was recently investigated and compared to CHX by Mahyari et al. The polyherbal mouthwash had similar effectiveness to CHX [576].

## 5. Constraints

Various factors, including anecdotes, cultural practices, societal repercussions, inaccurate information about health, cost, availability, lucrative businesses, innovative advertising, and consumer choice, influence the use of herbal supplements. Natural medicine is used widely, irrespective of geographical and demographic differences. However, the encouraging outcomes of most herbal supplements in contemporary treatment are few, conflicting, or inconsistent [577]. Instead, there is more evidence that herbal supplements may have potentially harmful effects, leading to public health concerns [578].

Several promising studies have shown the efficacy of herbal products in treatments; however, many of them have not been tested and are relatively unmonitored [578]. Due to the lack of knowledge regarding their modes of action, potential adverse effects, contraindications, and interactions with conventional pharmaceuticals and foods, it would not be easy to establish a rationale for the safe usage of these agents. It is, therefore, challenging to determine which therapies are the safest and most effective. Herbal products could also be unsafely promoted due to inadequate quality controls, labeling, and patient education. A regulatory flaw exists regarding herbal products, as premarketing approval is not required by the Food and Drug Administration [326], unlike the situation for conventional pharmaceuticals such as prescription and OTC treatments [577]. Regulatory authorities must establish appropriate measures to protect public health, as safety remains a significant concern with traditional remedies. A safe and adequate quality of all-natural medicines is essential to accomplish this goal.

On the other hand, despite frequent public use of herbal medications, communication between physicians and patients is still limited. Physicians generally have limited knowledge of herbal supplements, and patients frequently conceal or underreport their usage of herbal supplements out of concern that their doctors would not be interested [579]. In addition, practitioners usually emphasize potential toxicities one-sidedly, even though determining the toxic properties of natural preparations is often challenging because patients usually self-medicate and are reluctant to disclose their information.

Most adverse reactions associated with plant-based products are associated with hepatotoxicity [580], often from the simultaneous use of other hepatotoxic agents, such as acetaminophen and nonsteroidal anti-inflammatory agents or hepatotoxic botanical components [581]. However, no toxic effects were reported by the reviewed investigations in this study. The list of medicinal plants presented here were declared safe according to the corresponding investigations, possibly due to the nature of the natural compounds and their low dosage in different oral hygiene aids, such as mouthwashes, toothpaste, chewing gums, gels, and patches. However, evidence concerning the safety of herbal-based oral hygiene aids in dentistry is still unsatisfactory.

Physicians and healthcare providers must increase their knowledge of these products, pharmacokinetics, and potential interactions with conventional medications. There should not be generalizations regarding herbal supplements’ effectiveness and safety in society. By cooperating with educational institutions, scientific organizations, mainstream media, and legislators, healthcare professionals can significantly contribute to increasing herbal supplements’ overall safety. Additional clinical research, formal education, legislative modifications, and international partnerships are essential for reducing the overall burden of herbal supplements in clinical settings.

## 6. Conclusions

This paper aimed to review the currently available information on the effects of herbal drugs used as supplements or treatments for periodontitis. According to the review’s findings, herbal medicine is an effective alternative to contemporary medicine. There is an abundance of evidence that pure phytochemicals, essential oils, and plant extracts have the potential to be converted into medications that can be used to treat or prevent periodontitis. More studies on the safety and efficacy of these products are required to ascertain whether they have medicinal value, either alone or in combination with conventional treatment options, which can help reduce the overall burden of oral diseases globally, although the numerous clinical trials for these products are encouraging.

## Figures and Tables

**Table 1:** Clinical trials studying plant-based antimicrobials in periodontal diseases.

Natural Compound	Study Type	Samples Studied	Methods	Result(s)/Conclusion(s)	Limitations	Ref./Year
*Acacia arabica*	RCT	Nc: 40 Ns: 40 [Patients with mild–moderate periodontitis]	Gc: SRP + placebo Gs: SRP + *Acacia arabica* [PD and CAL were compared at baseline and after 15 and 90 days]	*Acacia arabica’s* antiplaque and antigingivitis properties were significantly valuable (*p* < 0.05). A reduction in sites with moderate PD was observed more among Gs than Gc (*p* = 0.001). The treatment may be prescribed with SRP for mild to moderate chronic periodontitis.	1. No assessment of bone defect fill. 2. Short follow-up period.	[56]/2018
	RCT	Nc: 30 Ns: 30 [Patients with gingivitis]	Gc: Regular toothpaste Gs: Toothpaste containing *Acacia arabica* [PI, GI, and BOP were compared at the baseline and after 28 days]	Gs showed statistically significantly better results regarding PI, GI, and BOP (*p* < 0.001). Gingivitis may be prevented with toothpaste that contains *Acacia arabica*. Using it daily can help improve oral health.	Not assessing the combination therapy using herbal toothpastes and mouthwashes.	[156]/2012
*Allium sativum*	RCT	Nc: 100 Ns: 100 [Patients with mild–moderate periodontitis]	Gc: Placebo tablets Gs: Tablets containing 300 mg of AGE powder [Subjects were examined at the start and 12 and 18 months post-op. GR and PD were measured]	The mean value of pocket depth was 1.06 ± 0.49 in comparison with the baseline value of 1.89 ± 0.74 (*p* < 0.001). The corresponding value was 1.50 ± 0.46 for the placebo group (*p* < 0.001). Periodontal disease can be prevented or improved with aged garlic extract.	Not determining the exact dosage, required duration of usage, or the principal mode of action.	[83]/2020
*Aloe barbadensis Miller*	RCT	N1: 10 N2: 10 [Patients with gingivitis]	G1: First, Aloe vera toothpaste for 14 days, then fluoride toothpaste for another 14 days G2: First, fluoride toothpaste for 14 days, then Aloe vera toothpaste for another 14 days [PI and GI were assessed]	PI was 2.14 ± 1.3 at baseline and 1.84 ± 1.02 at 30 days (*p* < 0.098). GI was 0.62 ± 0.74 at baseline and 0.25 ± 0.46 at 30 days (*p* < 0.068). In comparison with fluoride toothpaste, Aloe vera toothpaste had similar effects on PI and GI, and it seems that it can be used as an alternative.	1. Small sample size 2. Short follow-up period	[157]/2021
	RCT	Nc: 20 Ns: 20 [Patients with chronic periodontitis]	Gc: SRP Gs: SRP + Aloe vera gel [GI, PI, and PD were evaluated at baseline and after 15 and 30 days]	The mean reduction in GI: baseline day, 1.98 ± 0.10; 15 days, 1.6 ± 0.10; 30 days, 1.05 ± 0.10. After and before treatment measurements. Aloe vera treatment significantly reduced PI. The plaque index was meaningfully decreased from 2.15 ± 0.271 to 1.60 ± 0.34 after 30 days. Periodontitis significantly decreased in areas treated with Aloe vera gel.	1. Small sample size 2. Short follow-up period	[158]/2019
	RCT	Nc: 18 Ns: 18 [Healthy individuals]	Gc: Close-up tooth gel Gs: Aloe vera tooth gel [After 3 weeks, PI and GI were measured at baseline]	It was demonstrated that toothpaste containing Aloe vera significantly improved GI and PI; the results were similar to those achieved with tooth gel applied close up.	1. Small sample size 2. Short follow-up period	[159]/2018
	RCT	N1: 130 N2: 130 N3: 130 [Patients with PI > 1.9, and GI > 1.1]	G1: Aloe vera mouthwash G2: CHX mouthwash G3: Placebo [GI and PI were measured at baseline and after 15 and 30 days]	Compared to the CHX and Aloe vera groups, all parameters presented considerable reductions (*p* < 0.05). Neither Aloe vera nor CHX presented a significant difference (*p* < 0.05). Aloe vera and placebo and CHX and placebo had significantly different mean PI and GI scores (*p* < 0.000).	Short follow-up period	[160]/2016
	RCT	N1: 30 N2: 30 N3: 30 [Healthy individuals]	G1: Aloe vera mouthwash G2: CHX mouthwash G3: Placebo (normal saline) [After 15 and 30 days, GI and PI were evaluated at baseline]	Both Aloe vera and CHX significantly reduced plaque and gingivitis. There was no statistically significant difference between groups (*p* > 0.05). CHX mouthwash can be replaced with Aloe vera as an affordable and suitable alternative.	1. Small sample size 2. Short follow-up period	[161]/2016
	RCT	N1: 30 N2: 30 N3: 30 [Patients with visible plaque and gingivitis in at least 30% of their teeth]	G1: Aloe vera mouthwash G2: Chlorine dioxide mouthwash G3: CHX mouthwash [GI and PI were evaluated at baseline and after 15 days]	At follow-up, all three groups had significantly lower plaque and gingival scores than at baseline (*p* < 0.001). In comparison with Aloe vera, CHX significantly reduced PI and GI (*p* < 0.05).	1. Small sample size 2. Short follow-up period	[162]/2016
	RCT	N1: 30 N2: 30 N3: 30 [Patients with periodontitis]	G1: 0.2% CHX mouthwash G2: Green tea–Aloe vera mouthwash G3: Distilled water [After 14 days, GI, BI, and PI were evaluated at baseline]	G1, G2, and G3 reduced the PI by 0.17 ± 0.14, 0.10 ± 0.08, and 0.02 ± 0.18, respectively (*p* = 0.008). Between G1 and G2 with G3 were a significant difference. BIs between these three groups were significantly different, with *p* = 0.001 between G2 and G1 with G3. Periodontal health was improved by green tea–Aloe vera mouthwash. This can lead to improved dental and oral health.	1. Small sample size 2. Short follow-up period	[163]/2016
	RCT	N1: 115 N2: 115 N3: 115 [Healthy individuals with baseline DMFT index of 2.5 to 5 and baseline PI > 1.5]	G1: Mouthwash containing Aloe vera G2: CHX mouthwash G3: Placebo mouthwash [After 15 and 30 days, GI and PI were evaluated at baseline and]	CHX and Aloe vera groups reduced plaque and gingivitis significantly, but there was no statistically significant difference (*p* > 0.05). The ability of Aloe vera to reduce periodontal indexes makes it an effective mouthwash.	Short follow-up period	[164]/2014
	RCT	N1: 40 N2: 40 N3: 40 [Healthy individuals]	G1: 100% Aloe vera mouthwash G2: CHX mouthwash G3: Placebo mouthwash [At 7, 14, and 22 days, GI, BI, and PI were evaluated at baseline]	The PI, GI, and BI scores of G1 and G2 decreased statistically significantly after rinse regimens were initiated compared with G3. Aloe vera mouthwash significantly decreased gingivitis and plaque, but not as much as CHX.	1. Canceling regular oral hygiene was an inconvenient and embarrassing prerequisite in this mouth rinse study. 2. Short follow-up period	[100]/2012
	RCT	Nc: 20 Ns: 20 [Patients with chronic periodontitis]	Gc: SRP Gs: SRP + local administration of Aloe vera gel [GI, PI, and PD were measured at baseline and after 30 and 60 days]	There was no statistically significant difference between the control and experimental groups in PI in any of the three stages. Both groups treated with SRP combined with Aloe vera or SRP indicated substantial improvement in all 3 stages regarding GI and PD. As a result, Gs had significantly lower GI than the control group (*p* = 0.0001) and PD (*p* = 0.009).	1. Small sample size 2. Short follow-up period	[165]/2017
*Berberis vulgaris*	RCT	N1: 25 N2: 10 N3: 10 [Patients were healthy dormitory students]	G1: Barberry gel G2: Placebo gel without an active ingredient G3: Colgate^®^ antiplaque toothpaste [GI and PI were measured at baseline and after 21 days]	Between placebo and barberry gel groups, Colgate^®^ and placebo groups, there were significant differences in PI and GI (*p* < 0.01). However, there was no statistically significant difference between barberry and Colgate^®^ groups. By applying barberry dental gel, school-aged children can effectively control microbial plaque and gingivitis.	1. Lack of randomization 2. Small sample size 3. Lack of blinding 4. Short follow-up period	[166]/2007
*Camellia sinensis*	RCT	Nc: 15 Ns: 15 [Patients with generalized gingivitis]	Gc: Full-mouth prophylaxis Gs: Green tea extract and oral lycopene for 45 days + oral prophylaxis. [BI, salivary UA, and PI levels were measured at baseline and after 45 days]	After treatment, a comparison of the test and control groups revealed statistically significant results in BI (*p* ≤ 0.001), salivary UA levels (*p* ≤ 0.01), and PI (*p* ≤ 0.001). Gingivitis can be treated with lycopene and green tea extract as adjunctive prophylactic and therapeutic methods.	1. Small sample size 2. Short follow-up period	[167]/2019
	RCT	Nc: 15 Ns: 15 [Patients with chronic periodontitis]	Gc: No intervention Gs: Green tea herbal [After six weeks, BI, PI, and PD were evaluated at baseline]	Before and after SRP, both groups presented significant reductions in PD and BI; the intervention group presented a greater reduction (*p* = 0.003 and 0.031, respectively). The effect of reducing PI between the two groups was not significant, despite being significant within each group (*p* = 0.135). According to this study, green tea herbal may effectively treat periodontal diseases and improve the benefits of phase I periodontal therapy.	1. Lack of blinding 2. Small sample size 3. Lack of randomization 4. Short follow-up period	[168]/2018
	RCT	Nc: 20 Ns: 20 [Patients with a gingival index ≥ 1]	Gc: 0.12% CHX mouthwash Gs: Green tea mouthwash with 1% tannin [BI, PI, GI, and staining were measured at baseline and after one and four weeks]	After 1 and 4 weeks, significant differences were detected between groups, but not between groups, in all indices (*p* < 0.0001). Significantly less tooth staining was observed with the test mouthwash than with the control mouthwash. An adjunct to mechanical plaque reduction could be 1% tannin green tea mouthwash.	1. Small sample size 2. Observation bias (Hawthorne effect) 3. Short follow-up period	[169]/2017
	RCT	Nc: 22 Ns: 23 [Patients with marginal gingivitis]	Gc: Placebo gum Gs: Chewing gum containing green tea [BI, PI, and salivary IL-1β were measured at baseline and after 7 and 21 days]	There was a significant impact of chewing gum on BI and PI (*p* < 0.001). BI and PI mean changes at different observation periods were significantly different between the two groups (*p* < 0.001). Chewing gum had a significant effect on IL-1 β (*p* < 0.001). A significant difference in mean IL-1 β changes within 1–21 days was not observed (*p* = 0.086).	1. Small sample size 2. Short follow-up period	[170]/2016
	RCT	Nc: 55 Ns: 55 [Patients with PI and GI of at least 1.5 and 1, respectively]	Gc: Placebo mouthwash Gs: Mouthwash containing 2% green tea [GI and PI were measured at baseline and after 28 days]	Between baseline and 28 days, mean GI and PI scores decreased significantly among the test group, but not in the control group (*p* < 0.05). GI scores in the test group (0.67 ± 0.22) were statistically significantly reduced as compared with the control group (0.05 ± 0.11), and PI scores (1.65 ± 0.68) were statistically significantly reduced as compared to the control group (0.45 ± 0.99).	1. Short follow-up period 2. Small sample size	[171]/2015
	RCT	N1: 20 N2: 20 N3: 20 [Patients were healthy dormitory students]	G1: 0.2% CHX G2: 0.05% sodium fluoride G3: 0.5% *Camellia sinensis* extract [Salivary pH, PI, GI, and OHI scores were measured at baseline and after one and two weeks]	The experimental groups showed a reduction in mean PI and GI over the 2-week trial period. In all groups, antiplaque effectiveness was highest in G3 (*p* < 0.05). G1 and G3 were more effective than sodium fluoride at cleaning gingiva (*p* < 0.05). As compared to G1, the salivary pH increased in G2 and G3. In G1 and G3, the improvement in oral hygiene was more apparent. Due to its minimal side effects and prophylactic benefits, *Camellia sinensis* can be utilized as an adjunct to oral self-care.	1. Small sample size 2. Short follow-up period	[172]/2015
	RCT	Nc: 25 Ns: 25 [Patients with chronic gingivitis]	Gc: Normal saline Gs: Mouthwash containing 5% green tea [After five weeks, GI, PI, and BI were measured at baseline]	The periodontal indices showed significant improvement throughout this study (*p* < 0.001). The changing alteration patterns of indices were contrasted between two groups (*p* < 0.05). Even though the mouthwash group showed a greater overall improvement, the differences did not reach statistical significance (*p* > 0.05, observed power for GI: 0.09, PI: 0.11, and BI: 0.07). Green tea mouthwash is effective and safe for treating inflammatory periodontal diseases.	1. Short follow-up period 2. Small sample size	[173]/2012
	RCT	Nc: 10 Ns: 10 [Patients with chronic periodontitis]	Gc: SRP Gs: SRP + green tea catechin application [GI, PI, and PD were measured at baseline and after one and five weeks. After subgingival plaque sampling, red-complex bacteria were studied via PCR]	Between baseline and 1 week and baseline and 5 weeks, both study and control groups showed significant differences in PD, GI, and PI and substantial reductions in red-complex organisms. (*p* < 0.001). PD, PI, and GI did not show statistically significant intergroup differences. In Gs, *T. forsythus* was significantly reduced at one week and five weeks and *P. gingivalis* was significantly reduced at one week compared to Gc. Chronic periodontitis can be effectively treated with green tea catechins in combination with SRP.	1. Short follow-up period 2. Split-mouth design 3. Small sample size	[174]/2013
*Citrus sinensis*	RCT	Nc: 10 Ns: 10 [Patients with moderate-severe gingivitis]	Gc: 2% CHX mouthwash Gs: *Citrus sinensis* (4% ethanolic extract) mouthwash [GI, PI, and BI were measured at baseline and after 7 and 14 days]	A mouthwash containing *citrus sinensis* 4% reduced PI (*p* = 0.095) as well as a mouthwash containing CHX 0.2% and BI (*p* = 0.42). However, the extract was more efficient in lowering GI (*p* = 0.04).	1. Lack of blinding 2. Small sample size 3. Lack of randomization 4. Short follow-up period	[109]/2018
*Curcuma longa*	RCT	Nc: 15 Ns: 15 [Patients with chronic periodontitis]	Gc: SRP + CHX gel Gs: SRP + curcumin gel [After 30 and 45 days, GI, PI, BI, and PD were evaluated at baseline]	A significant difference in PI, PD, BI, and GI was found between Gs and Gc (*p* < 0.001). SRP can be administered with the control and experimental gel, but curcumin gel performed better than CHX gel in reducing periodontal pockets in mild to moderate cases.	1. Short follow-up period 2. Lack of microbiological evaluations 3. Small sample size	[175]/2016
	Pilot Study	Ten patients with severe gingivitis	Curcuma gel was consumed orally by the subjects for 21 days; BI was measured after three weeks	Statistical significance was found in the results (*p* < 0.001). By reducing gingival inflammation, Curcuma longa extract gel was effective.	1. Short follow-up period 2. Pilot study 3. Small sample size	[176]/2014
*Garcinia mangostana*	RCT	Nc: 25 Ns: 25 [Patients with generalized chronic periodontitis]	Gc: SRP + placebo gel Gs: *mangostana* gel + SRP [At baseline and after three months, PI, BI, PD, CAL, and red-complex bacteria were evaluated]	Between baseline and three months after the study began, Gs had significantly lower PD, CAL, BI, PI, and *Treponema denticola* values than the placebo group (*p* ≤ 0.05).	1. Small sample size 2. Short follow-up period	[177]/2017
*Glycyrrhiza glabra*	RCT	Nc: 52 Ns: 52 [Patients with mild–moderate periodontitis]	Gc: No intervention Gs: 10% *G. glabra* gum paint [GI, PD, and CAL were evaluated at baseline and after four weeks]	Patients in the study group showed significant improvements in their periodontal health. *G. glabra* prevented periodontal diseases.	1. Small sample size 2. Short follow-up period	[178]/2019
*Juglans regia*	RCT	N: 20 [Patients with mild gingivitis]	2% and 3% ether extracts of *Juglans regia*, 2% and 3% petroleum extracts of *Juglans regia*, 2% water-soluble extract of *Juglans regia*, and propylene glycol vehicles were evaluated. [PI was measured at baseline and after three days]	There was 32.12% and 31.56% antiplaque activity in 2% and 3% ether fractions of Juglans regia, respectively. Inhibition of plaque was observed in 30.32% of cases with the 2% aqueous solution of Juglans regia, and in 17.62% and 19.45% of cases with the 2% and 3% petroleum ether fractions. A high level of statistical significance was found in all the findings. The researchers concluded that Juglans regia could be used as an adjunct to oral hygiene regimens since it displayed potent anti-plaque properties.	1. Small sample size 2. Short follow-up period	[179]/2009
*Lippia sidoides*	RCT	N1: 10 N2: 10 N3: 10 [Healthy individuals]	G1: *Lippia sidoides* EO G2: CHX G3: Placebo [PI and BI were measured at baseline and after three months]	In the test groups, plaque and gingivitis were significantly reduced (*p* < 0.05), Statistically, there was no significant differences (*p* > 0.05). Gel preparations containing *Lippia sidoides* essential oil were effective against plaque and gingivitis.	1. Small sample size 2. Short follow-up period	[180]/2013
	RCT	Nc: 28 Ns: 27 [Patients with PI and GI of at least 1.5 and 1, respectively]	G1: CHX mouthwash G2: *Lippia sidoides* EO mouthwash [PI, BI, GI, and salivary *S. mutans* colony counts were measured at baseline and after 7 and 30 days]	Clinical and microbiological parameters were significantly reduced by both mouth rinses. Both groups showed no significant differences (*p* > 0.05). Both groups had considerable reductions in the number of colonies of *S. mutans* (*p* < 0.05). Although CHX treatment reduced more efficiently than *L. sidoides*, there was no statistical difference between the two groups, and both treatments reduced the bacteria equally (*p* = 0.3). The results of this study demonstrate that *Lippia sidoides* EO mouth rinses reduce gingival inflammation and microbial plaque.	1. Small sample size 2. Short follow-up period	[181]/2009
*Mangifera indica*	RCT	Nc: 10 Ns: 10 [Healthy individuals]	Gc: CHX mouthwash Gs: Mango leaf mouthwash [After five days, PI, GI, and salivary *S. mutans*, *S. mitis*, and *S. salivarius* counts were evaluated at baseline]	Mango leaf and CHX mouthwashes significantly reduced the microbial count and improved gingival health and plaque control, with CHX showing a greater reduction in the microbial count and better plaque control.	1. Small sample size 2. Short follow-up period	[182]/2017
Manuka honey	RCT	N1: 7 N2: 7 N3: 6 [Orthodontic patients]	G1: Honey G2: 10% sucrose G3: 10% sorbitol [Plaque pH, bacterial count, and antibacterial properties of honey against *S. mutans*, *L. acidophilus*, and *P. gingivalis* were measured at baseline and after 2, 5, 10, 20, and 30 min]	As compared to sorbitol, honey and sucrose had significantly different plaque pH values (*p* ≤ 0.001). Honey was the only substance that significantly reduced pH after 30 min of exposure, despite sucrose, sorbitol, and honey significantly reducing bacteria recovery (*p* < 0.001). There was a significant decrease in the growth of all bacterial strains when honey was added (*p* ≤ 0.001). By applying honey topically, pH can be modified, bacteria counts can be reduced, and bacterial growth can be inhibited.	1. No follow-up 2. Small sample size	[183]/2014
	Pilot study	Nc: 15 Ns: 15 [Healthy individuals]	Gc: Sugar-free chewing gum Gs: Manuka honey products [PI and GI were measured at baseline and after 21 days]	Manuka honey reduced plaque scores and bleeding sites (48% reduced to 17%; *p* = 0.001) statistically significantly compared to the control group. The results suggest that manuka honey may have therapeutic potential in treating gingivitis and periodontitis.	1. Short follow-up period 2. Pilot study 3. Small sample size	[184]/2004
*Matricaria chamomilla*	RCT	N1: 25 N2: 25 N3: 25 [Patients with chronic periodontitis]	G1: SRP + placebo G2: 0.12% CHX + SRP G3: 1% *Matricaria chamomilla* mouthwash + SRP [PI, BI, GI, PD, CAL, GR, stain index, and microbial colony counts were measured at baseline and after six weeks and three months]	All parameters (except GR in the placebo group) changed significantly between baseline and three months. Compared to the placebo group, meditative mouth rinses containing chamomilla exhibited significant benefits. In comparison to baseline, the CHX rinse resulted in slightly higher improvements in both PD (3.68 mm vs. 3.36 mm) and CAL (3.00 mm vs. 2.72 mm) than CHX rinse. Non-surgical periodontal therapy for chronic periodontitis can use *Matricaria chamomilla* as an adjunct to CHX mouthwash.	1. Small sample size 2. Short follow-up period	[185]/2020
	Pilot study	N1: 10 N2: 10 N3: 10 [Orthodontic patients with fixed appliances]	G1: Placebo G2: 0.12% CHX G3: 1% *Matricaria chamomilla* mouthwash [PI and BI were measured at baseline and after 15 days]	G1 exhibited increases in PI and BI (10.2% and 23.1%, respectively). The PI and BI levels in G3(−25.6% and −29.9%, respectively) and G2 (−39.9% and −32.0%, respectively) were considerably lower than those in the placebo group. Biofilm formation and BI were reduced in gingivitis patients. This was probably a result of *Matricaria chamomilla*’s anti-inflammatory and antimicrobial properties	1. Short follow-up period 2. Pilot study 3. Small sample size	[186]/2016
*Psidium guajava*	RCT	N total: 15 patients (30 sites) Nc: 15 sites Ns: 15 sites [Patients with chronic periodontitis]	Gc: SRP Gs: 3% *P. guajava* gel [After one and three months, PI, GI, BI, PD, CAL, and colony counts of *A. a* and *P. gingivalis* were evaluated at baseline]	Clinical parameters improved significantly throughout the study. Three months after testing, site-specific indices, PD (2.74 ± 0.283), and CAL (2.8 ± 0.152) showed statistically significant reductions. *A. a*. (17.4 ± 0.026) and *P. gingivalis* (22.7 ± 1.225) colony counts were significantly reduced at one and three months in the test sites (*p* < 0.001). Local delivery of 3% *P. guajava* gel treated chronic periodontitis with clinical and microbiological parameter improvements.	1. Short follow-up period 2. Small sample size 3. Split-mouth design	[187]/2021
*Punica granatum*	RCT	N total: 10 patients (20 sites) Nc: 10 sites Ns: 10 sites [Patients with moderate–severe chronic periodontitis]	Gc: Placebo gel Gs: *Punica granatum* gel [after 15 days, PI, GI, and BI were measured at baseline]	After 15 days following gel application, mean BI, GI, and PI were significantly reduced. According to microbiological results, *Punica granatum* oral gel suppresses microbial growth. Test specimens revealed mild perivascular inflammation and increased collagen fibers, while controls showed dense inflammatory infiltration and collagen fiber destruction. In combination with SRP, *Punica granatum* gel reduced chronic periodontitis clinical symptoms.	1. Small sample size 2. Short follow-up period	[188]/2019
	RCT	N1: 20 N2: 20 N3: 20 N4: 20 [Healthy individuals]	G1: Pomegranate extract gel G2: CHX gel G3: Ornidazole–CHX gel G4: Placebo gel [After 14 and 60 days, GI, PI, BOP, PD, and GCF levels of IL-8, IL-1β and chemokine ligand 28 were measured at baseline]	Inhibition of inflammatory cytokines and chemokines was observed in G1. G1 levels of IL-1β and IL-8 increased less than CCL28 levels (*p* = 0.003, 0.002), which remained unchanged from baseline (*p* = 0.15). G1 subjects showed a lower increase in BOP and GI (*p* = 0.01, 0.05) compared to other groups (*p* < 0.001) after 14 days. It was similar in terms of PI reduction between G1 and G3 gels (*p* = 0.96). For the treatment of gingivitis, PEG effectively reduced inflammatory markers.	1. In an experimental gingivitis model, all products were tested, which may differ from the natural gingivitis model. 2. In order to avoid bias caused by variable host responses, a cross-over design would have been more appropriate.	[189]/2017
	RCT	Nc: 40 Ns: 40 [Diabetic patients with gingivitis]	Gc: CHX 0.2% Gs: *Punica granatum* mouthwash [After 14 days, PI, GI, BI, and PD were measured at baseline]	Both interventions significantly improved gingival and plaque indices (*p* < 0.001 for all indices). Primary outcome measures showed no significant differences between Gc and Gs, except for GI, where Gs mouthwash had superiority over Gc after two weeks (*p* = 0.039). It is safe and effective to use *Punica granatum* mouthwash as an alternative to CHX for diabetic patients with gingivitis.	1. Short follow-up period 2. Lack of a placebo group 3. Small sample size	[190]/2016
*Rosmarinus officinalis*	RCT	Nc: 23 Ns: 23 [Patients with moderate chronic periodontitis]	Gc: SRP + placebo Gs: SRP + EO mouth rinse [After three and six months, PD, CAL, BOP, and BI were evaluated at baseline; the subgingival plaque was sampled to evaluate principal periodontitis-associated bacteria]	A significant improvement in CAL was observed after 3 and 6 months compared to the control group (*p* < 0.001). Following SRP, adding essential oils to mouthwashes decreases subgingival bacterial levels and improves clinical outcomes.	Small sample size	[191]/2016
*Salvadora persica*	RCT	Nc: 47 Ns: 47 [School students]	Gc: Fluoridated toothpaste + brushing Gs: SP sticks [Baseline, three-week, and 12-week PI measurements were conducted as well as saliva sampling]	Plaque scores decreased statistically significantly in both groups (*p* = 0.007 and *p* = 0.001, respectively). After three months, the number of subjects with abundant *S. sanguinis* increased from zero to six.	1. Small sample size 2. Short follow-up period	[192]/2020
	RCT	N total: 44 [Pediatric patients receiving chemotherapy]	Gc: Normal saline Gs: Persica oral drops [Oral conditions were recorded at baseline and after 8 and 15 days]	A comparison of the severity of mucositis and oral health status of patients in both examination sessions did not reveal significant differences between treatment groups (*p* > 0.05). Mucositis, plaque accumulation, and gingival health improved statistically significantly in both treatment groups after 14 days following mouth rinse administration (*p* < 0.05). SP oral drops significantly improve plaque and gingival health	1. Small sample size 2. Short follow-up period	[193]/2020
	RCT	N1: 12 N2: 13 [Patients with grade two or three plaque score]	G1: Toothpaste with tea tree oil G2: Miswak-based toothpaste [PI was recorded at baseline and after 24 h of follow-up]	Both herbal-based toothpastes reduced plaque scores, but when compared with G1, G2 resulted in significantly lower plaque scores.	1. Short follow-up period 2. Lack of gingival inflammation assessment 3. Small sample size	[194]/2018
	Cross-sectional	N1: 115 N2: 93 N3: 79	G1: SP sticks (Miswak) G2: Conventional toothpaste/toothbrush G3: SP sticks + toothbrush [GI, OHI, and PI were recorded]	G1 had a higher mean GI than G2, and G3 had a lower mean PI than G2. Between G1 and G2, the mean GI score was statistically significant (*p* = 0.001). Oral hygiene did not differ statistically significantly between groups.	Small sample size	[195]/2012
*Terminalia chebula*	RCT	N1: 30 N2: 30 N3: 30 [Healthy students]	G1: *T. chebula* mouthwash G2: CHX G3: Distilled water At baseline and after 15 and 30 days, PI and GI were evaluated]	At 15 and 30 days, PI and GI decreased significantly in G1 and G2 (*p* < 0.05). There was a significant reduction in G2, but not statistically significant in comparison to G1. The GI between G1 and G2 was not statistically significant (*p* = 0.837 for 15 days and *p* = 0.909 for 30 days) and PI (*p* = 0.592 at 15 days and *p* = 1.096 at 30 days). Using the *T. chebula* mouth rinse reduced dental plaque and gingivitis as effectively as CHX without the adverse effects of CHX.	1. Small sample size 2. Short follow-up period	[196]/2015
	RCT	N1: 26 N2: 26 N3: 26 [Patients with PI > 1.5]	G1: 0.12% CHX G2: *Terminalia chebula* 10% mouthwash G3: Saline rinse [At baseline and after 7 and 14 days, PI and GI were evaluated]	Clinical parameters were significantly reduced in both G1 and G2 even though there were no significant differences between them (*p* > 0.05). Studies have shown that *Terminalia chebula* mouth rinses reduce microbial plaques and gingival inflammations, as well as neutralize salivary pH levels.	1. Small sample size 2. Short follow-up period	[197]/2014
	RCT	Nc: 40 Ns: 40 [Patients with chronic gingivitis]	Gc: Oral prophylaxis alone Gs: Oral prophylaxis + gingival massage with *T. chebula* powder [At baseline and after one month, PI, GI, and BI were measured]	Significant reductions in the PI, GI, and BI scores were observed after gum massage with *T. chebula* powder. Chronic gingivitis patients can benefit from *T. chebula* powder.	1. Short follow-up period 2. No comparison with other studies 3. Small sample size	[198]/2017

**RCT:** Randomized Clinical Trial; **CAL:** Clinical Attachment Level; **Nc:** Number of Subjects in the Control Group; **Ns:** Number of Subjects in the Study Group; **Gc:** Control Group; **Gs:** Study Group; **GI:** Gingival Index, **GR**: Gingival Recession; **AGE:** Aged Garlic Extract; **SRP:** Scaling and Root Planning; **PD:** Pocket Depth; **CHX:** Chlorhexidine; **BI:** Bleeding Index; **UA:** Uric Acid; **OHI**: Oral Hygiene Index; **BOP:** Bleeding on Probing; **PCR:** Polymerase Chain Reaction; **EO:** Essential Oil; **GCF:** Gingival Crevicular Fluid; **PI:** Plaque Index, **SP:** *Salvadora persica.*

## Data Availability

This article is a review and does not contain any studies with humans or animals performed by the authors.

## Abbreviations

**AC-PACs:** A-Type Cranberry Proanthocyanidins; **AGE:** Aged Garlic Extract; **BBR:** Berberine; **BI:** Bleeding Index; **BID:** Twice Daily; **BOP:** Bleeding on Probing; **CAL:** Clinical Attachment Level; **CBEO:** *Cinnamomum* Bark Essential Oil; **CFU:** Colony-Forming Unit; **CHX:** Chlorhexidine; **CR:** *Coptidis rhizoma*; **CS:** *Camellia sinensis*; **EO:** Essential Oil; **GE:** Garlic Extract; **GI:** Gingival Index; **GSE:** Grape Seed Extract; **LPS:** Lipopolysaccharide; **MBC:** Minimal Bactericidal Concentration; **MDR:** Multidrug-Resistant; **MIC:** Minimum Inhibitory Concentration; **MMP:** Matrix Metalloproteinase; **MRSA:** Methicillin-Resistant *Staphylococcus Aureus;* **MTC:** *Matricaria chamomilla;* **NO:** Nitric Oxide; **PD:** Pocket Depth; **PDL:** Periodontal Ligament **PI:** Plaque Index; **PL:** *Pistacia lentiscus;* **PYC:** Pycnogenol^®^; **SP:** *Salvadora persica;* **SRP:** Scaling and Root Planning; **TID:** Three Times Daily; **VSC:** Volatile Sulfur Compounds.
